# Structural basis of TFIIH activation for nucleotide excision repair

**DOI:** 10.1038/s41467-019-10745-5

**Published:** 2019-06-28

**Authors:** Goran Kokic, Aleksandar Chernev, Dimitry Tegunov, Christian Dienemann, Henning Urlaub, Patrick Cramer

**Affiliations:** 1 Department of Molecular Biology, Max Planck Institute for Biophysical Chemistry, Am Fassberg 11, 37077 Göttingen, Germany; 2 Max Planck Institute for Biophysical Chemistry, Bioanalytical Mass Spectrometry, Am Fassberg 11, 37077 Göttingen, Germany; 3 University Medical Center Göttingen, Institute of Clinical Chemistry, Bioanalytics Group, Robert-Koch-Straße 40, 37075 Göttingen, Germany

**Keywords:** Cryoelectron microscopy, Nucleotide excision repair

## Abstract

Nucleotide excision repair (NER) is the major DNA repair pathway that removes UV-induced and bulky DNA lesions. There is currently no structure of NER intermediates, which form around the large multisubunit transcription factor IIH (TFIIH). Here we report the cryo-EM structure of an NER intermediate containing TFIIH and the NER factor XPA. Compared to its transcription conformation, the TFIIH structure is rearranged such that its ATPase subunits XPB and XPD bind double- and single-stranded DNA, consistent with their translocase and helicase activities, respectively. XPA releases the inhibitory kinase module of TFIIH, displaces a ‘plug’ element from the DNA-binding pore in XPD, and together with the NER factor XPG stimulates XPD activity. Our results explain how TFIIH is switched from a transcription to a repair factor, and provide the basis for a mechanistic analysis of the NER pathway.

## Abstract (web summary)

The NER machinery contains the multisubunit transcription factor IIH (TFIIH) that opens the DNA repair bubble, scans for the lesion, and coordinates excision of the damaged site. Here the authors resolve the cryo-electron microscopy structure of the human core TFIIH-XPA-DNA complex and provide insights into its activation.

## Introduction

Genomes are constantly threatened by DNA damage, but cells can remove a large variety of DNA lesions by nucleotide excision repair (NER)^1^. Mutations in NER factors compromise cellular fitness and cause human diseases, such as Xeroderma pigmentosum (XP), Cockayne syndrome, and trichothiodystrophy (TTD)^2,3^. The NER machinery is built around the multisubunit transcription factor IIH (TFIIH), which opens the DNA repair bubble, scans for the lesion, and coordinates excision of the damaged DNA single-strand fragment^1,4^. TFIIH consists of a kinase module and a core module that contains the ATPases XPB and XPD^5^.

Here we prepare recombinant human TFIIH and show that XPB and XPD are stimulated by the additional NER factors XPA and XPG, respectively. We then determine the cryo-electron microscopy (cryo-EM) structure of the human core TFIIH-XPA-DNA complex at 3.6 Å resolution. The structure represents the lesion-scanning intermediate on the NER pathway and rationalizes the distinct phenotypes of disease mutations. It reveals that XPB and XPD bind double- and single-stranded DNA, respectively, and that XPA forms a bridge between XPB and XPD to retain the DNA at the 5′-edge of the repair bubble. Biochemical data and comparisons with prior structures^6,7^ explain how XPA and XPG can switch TFIIH from a transcription factor to a DNA repair factor. During transcription, the kinase module inhibits the repair helicase XPD^8^. For DNA repair, XPA dramatically rearranges the core TFIIH structure, which reorients the ATPases, releases the kinase module, and displaces a “plug” element from the XPD pore that holds DNA. This enables XPD to move by ~80 Å, engage with DNA, and scan for the lesion.

## Results

### Recombinant human TFIIH

A mechanistic dissection of the NER machinery was thus far hampered because TFIIH was not available in large quantities. We therefore established protocols to prepare milligram amounts of recombinant human TFIIH core and kinase modules (“Methods”). The purified TFIIH core comprised seven subunits including the ATPases XPB and XPD, whereas the kinase module contained CDK7, cyclin H, and MAT1. From these two modules, we could reconstitute the complete ten-subunit TFIIH (Supplementary Fig. 1a).

### Biochemical characterization of TFIIH ATPases

**Fig. 1:**
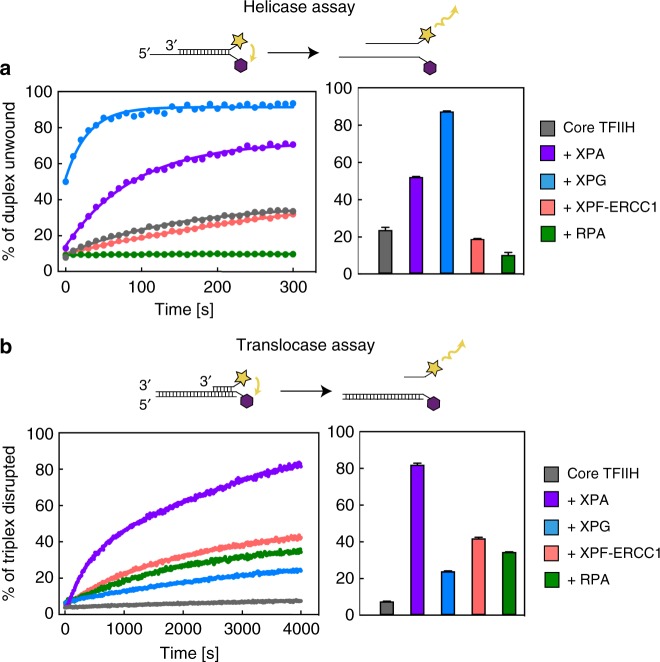
Regulation of ATPases in core transcription factor IIH. **a** Effect of nucleotide excision repair (NER) proteins on XPD 5′–3′ helicase activity. Real-time fluorescence measurement using a fluorescence energy transfer-based assay. Bars show the percentage of unwound product after 100 s (*n* = 2, error bars indicate the range of the data). RPA inhibits XPD helicase activity, by masking the single-stranded DNA overhang. Source Data are provided in the Source Data file. **b** Effect of NER proteins on XPB translocase activity. Real-time fluorescence measurement of triplex disruption. Bars show percentage of disrupted triplex after 4000 s (*n* = 2, error bars indicate the range of the data). Source Data are provided in the Source Data file

To analyze the enzymatic activities of TFIIH, we monitored helicase and translocase activities in real time by fluorescence resonance energy transfer (“Methods”). Core TFIIH showed 5′–3′ helicase activity, which was lost upon mutation of the XPD active site (Fig. 1a, Supplementary Fig. 1b). This is consistent with a prior description of XPD as a 5′–3′ DNA helicase^8,9^. Core TFIIH also showed translocase activity, which was however much lower than the helicase activity (Fig. 1b). Translocase activity was due to XPB because it was sensitive to triptolide (Supplementary Fig. 1c), a drug that targets XPB^10^. This is consistent with the known translocase activity for the yeast XPB homolog^11^ but not with helicase activity reported for an archaeal XPB homolog^12^.

### NER factors stimulate the TFIIH helicase XPD

To test whether other NER factors affect TFIIH activities, we purified human XPA, XPG, RPA, and XPF-ERCC1 complex (Supplementary Fig. 1d). In the presence of XPA or XPG, DNA unwinding by XPD was 4-fold or 20-fold faster, respectively, as deduced from stopped-flow kinetics (Fig. 1a, Supplementary Fig. 1e). A stimulation of XPD by XPA was observed before^9,13^, but the effect of XPG on DNA unwinding is much stronger. This explains the earlier observation that XPG is required for efficient DNA bubble opening^14^ and implicates XPG in lesion scanning by XPD. XPB translocation activity was stimulated by all NER factors tested, although stimulation by XPA was exceptionally strong (Fig. 1b).

### Structure of the core TFIIH-XPA-DNA complex

**Fig. 2:**
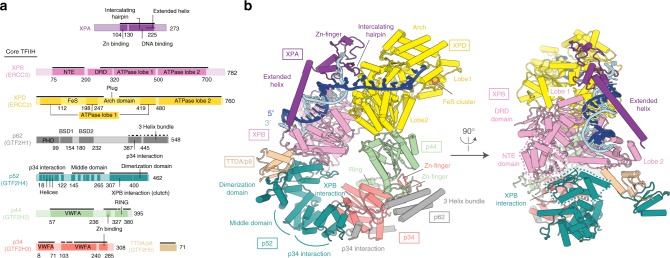
Structure of human core transcription factor IIH (TFIIH)-XPA-DNA complex. **a** Domain organization of XPA and human TFIIH subunits. Residues at domain borders are indicated. Solid and dashed black lines mark residues modeled as atomic and backbone structures, respectively. DRD damage recognition domain, NTE N-terminal extension, BSD BTF2-like, synapse-associated and DOS2-like domains, VWFA von Willebrand factor type A domain. **b** Cylindrical representation of the structure. Proteins colored as in **a**. Newly built XPB and p52 domains are highlighted with dashed lines and reveal how XPB is connected to the TFIIH core

We next investigated the structural basis for how XPA and XPG activate the TFIIH ATPases. We prepared the core TFIIH-XPA-XPG complex bound to a bifurcated DNA scaffold that mimics one half of a DNA repair bubble (Supplementary Fig. 2a). We imaged this complex by cryo-EM and solved the structure at an overall resolution of 3.6 Å (“Methods,” Supplementary Figs. 2 and 3). The cryo-EM density was of high quality and revealed DNA and all protein components except XPG, which likely dissociated during cryo-EM grid preparation. The derived structure contains the p52 subunit and other regions that were lacking from the previous human TFIIH structure^6^ and reveals the XPB-TFIIH core interface (Fig. 2, Supplementary Fig. 3f).

### TFIIH rearrangements

The structure of the core TFIIH-XPA-DNA complex differs substantially from the TFIIH structure observed in transcription complexes (Fig. 2)^7,15^. Both XPB and XPD bind DNA (Supplementary Fig. 4), whereas only XPB binds DNA in transcription complexes^7,15^. XPB binds DNA in the duplex region, whereas XPD binds the single-stranded 3′-DNA extension, consistent with translocase and helicase function, respectively. DNA binding of both ATPases requires large structural changes in TFIIH (Supplementary Movie 1). XPD and its associated subunit p44 move by ~80 Å, and this requires a flexible connection between subunits p44 and p34 and rearrangements in subunit p52 (Supplementary Fig. 5).

### XPA clamps TFIIH to DNA

The structure informs on XPA, which is essential for NER^16^. XPA contains an N-terminal zinc finger and a DNA-binding domain with an extended helix and an intercalating β-hairpin (Fig. 2). XPA forms an elongated arch over DNA that bridges between the two ATPases. XPA binds XPB and XPD with its extended helix and its intercalating hairpin, respectively. The C-terminal region of XPA extends to p52 and TTDA/p8 (Supplementary Figs. 3d and 7c), explaining why TTDA/p8 facilitates XPA recruitment in vivo^17^.

**Fig. 3:**
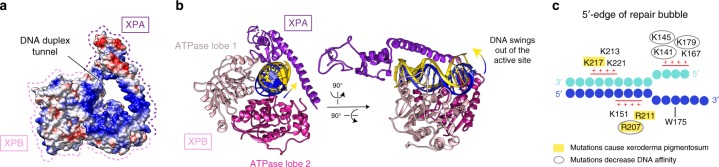
XPA–DNA interactions. **a** DNA duplex tunnel formed by XPA and XPB. Blue, white, and red color indicates positive, neutral, and negative electrostatic surface potential, respectively. Created with UCSF Chimera. **b** Two positions of DNA in the tunnel. Tightly bound DNA is in blue, dissociated DNA in yellow, ATPase lobe 1 of XPB in pink, ATPase lobe 2 in hot pink, and XPA in purple. **c** Electrostatic interactions between XPA and the DNA junction. DNA nucleotides are indicated as circles. Patches of positively charged residues in proximity to the DNA backbone are indicated. Residues that are mutated in Xeroderma pigmentosum are highlighted in yellow^16^. Mutation of encircled residues decreases DNA affinity^61^

These observations explain how XPA stimulates XPB translocation. First, XPA connects both XPB ATPase lobes to p52 and TTDA/p8 subunits that stimulate the XPB activity within the TFIIH core^18,19^. Second, XPB is not a processive enzyme and readily dissociates from DNA^11^. However, in our structure DNA is held in a positively charged DNA duplex tunnel that is formed between the extended helix of XPA and the XPB ATPase (Fig. 3a). XPA thus retains DNA near the XPB active site. Indeed, our cryo-EM data revealed an alternative state of the complex with the DNA duplex disengaged from XPB but retained by the XPA extended helix (Fig. 3b, Supplementary Fig. 2d). Thus, by trapping the DNA within the duplex tunnel, XPA may retain the NER machinery on the DNA during lesion scanning and processing.

### XPA demarcates the edge of the DNA repair bubble

XPA also contributes to the recognition of the 5′-edge of the DNA repair bubble that depends on electrostatic interactions (Fig. 3c). XPA inserts its intercalating β-hairpin between DNA single strands at the duplex junction (Fig. 2b), consistent with the published biochemical data^20^. The tip of the XPA hairpin contains a conserved tryptophan residue (Trp175) that stacks against the base of the DNA 3′-extension at the junction (Supplementary Fig. 3i). Several sites of mutations that cause severe XP^16^ map to XPA residues that interact with DNA (Fig. 3c). Previous studies of the yeast XPA counterpart suggested that XPA may detect DNA lesions^21^. However, our results suggest that XPA rather demarcates the 5′-edge of the repair bubble and stimulates lesion scanning by clamping core TFIIH onto DNA.

### Disease-related XPD mutations

The detailed structure also enables the localization of XPD residues mutated in patients with XP and TTD and rationalizes their functional effects, as previously suggested by biochemical studies^22^ and comparisons to archaeal XPD homologs in the absence of DNA^23–25^ (Supplementary Fig. 6, Supplementary Note 1). Most notably, the XP mutations affect the DNA-contacting residues (Supplementary Fig. 6b) in the ATPase lobe 2, which would specifically impair the XPD helicase activity and DNA repair. In contrast, TTD mutations map to the XPD–p44 interface, around the FeS cluster and between the XPD ATPase lobes (Supplementary Fig. 6c–e). Thus TTD mutations would compromise the integrity of TFIIH and XPD stability, thereby affecting transcription and other TFIIH-mediated processes outside NER^5^, which leads to more severe phenotypes.

### XPD–DNA interactions

**Fig. 4:**
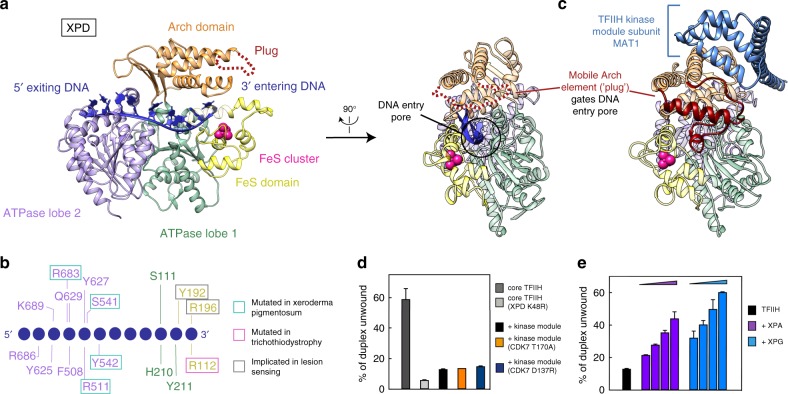
XPD activation and DNA binding. **a** Two views of XPD bound to DNA. XPD domains ATPase lobe 1, FeS, Arch, and lobe 2 are in green, yellow, orange, and medium purple, respectively. DNA is dark blue. A black circle depicts the DNA pore. **b** Schematic representation of XPD–DNA interactions. **c** Side view of XPD bound to the kinase module (PDB code 5OF4)^6^. The plug in the Arch domain is in dark red, the kinase module subunit MAT1 in blue. **d** Effect of kinase module variants on XPD helicase activity. Core transcription factor IIH (TFIIH) was incubated with two-fold excess of the kinase module and helicase activity was monitored as in Fig. 1a. Bars show the percentage of unwound product after 300 s (*n* = 2, error bars indicate the range of the data). Source Data are provided in the Source Data file. **e** Effect of increasing concentrations of XPA and XPG on XPD helicase activity in the presence of kinase module (0.25 μM core TFIIH, 0.5 μM kinase module, 0.375, 0.75, 1.5, or 3 μM XPA or XPG). Otherwise as in **d**. Source Data are provided in the Source Data file

The structure also provides details of the XPD–DNA interactions (Fig. 4a, b). The ATPase lobe 2 interacts with DNA bases near the duplex-single-strand junction, which includes base stacking with the side chains of residues F508 and Y627. This mode of DNA interaction is unusual for helicases of the SF2 family, which generally engage with the sugar-phosphate backbone^26,27^. We speculate that the extensive contacts of XPA and XPD with single-stranded DNA facilitate DNA opening and XPD loading during initial stages of NER^28^.

The structure further suggests how XPD verifies the lesion during DNA scanning. The DNA single strand extends into a pore formed by the ATPase lobe 1, the iron-sulfur cluster (FeS) domain, and the Arch domain (Fig. 4a). The sugar-phosphate backbone is bound by residues in the FeS domain, including Y192 and R196, which were implicated in DNA lesion sensing^29^ (Fig. 4b). Residues R112 and C134 bridge between DNA and the FeS cluster (Supplementary Fig. 4d), which was suggested to be involved in lesion detection via DNA-mediated charge transfer^30^. The FeS cluster is flanked by two protein pockets that are lined with aromatic residues Y158, F161, and F193 and may proof-read DNA bases, as observed for base excision DNA repair^31^.

### Mechanism of XPD repression

We could reproduce the known repression of the XPD helicase activity by the TFIIH kinase module^8,9^ in our helicase assays (Fig. 4d). We found that catalytically inactive variants of the TFIIH kinase module (Supplementary Fig. 1f) could also repress XPD helicase activity, showing that repression does not require the kinase activity of CDK7 (Fig. 4d). Both XPA and XPG could relieve the kinase-mediated repression of XPD in a concentration-dependent manner (Fig. 4e). These observations show that XPA and XPG counteract the repressive effect of the kinase module on XPD.

A comparison of our structure with the previous TFIIH structures shows how the kinase module represses XPD activity. In previous TFIIH structures^6,7^, a region in the XPD Arch domain forms a “plug” (residues 273–325) that occupies the DNA pore of XPD (Fig. 4c). The plug would clash with DNA in the XPD pore but is displaced and mobile in our structure (Fig. 4a). The kinase module subunit MAT1 contacts the plug and may stabilize it in the XPD pore, explaining how the kinase module impairs binding of core TFIIH to single-stranded DNA and XPD helicase activity^9^. In addition, a loop in the yeast counterpart of p62 extends into the XPD active site^7^ and would interfere with the observed DNA trajectory through the helicase.

### Mechanism of XPD activation by XPA

Structural comparisons also suggest how XPA relieves XPD inhibition by the kinase module. XPA stabilizes TFIIH in a new conformation in which the two ATPases are drastically reoriented. This conformation is incompatible with MAT1 binding as observed in the previous TFIIH structure^6^ (Supplementary Fig. 5e). This also explains how XPA facilitates kinase module removal upon NER induction in vivo^32^. Taken together, MAT1 and XPA stabilize two entirely different conformations of TFIIH, which contain the repair helicase XPD in an inactive or an active state, respectively.

### XPG in lesion scanning

Since XPG was not visible in our structure, we located it by chemical crosslinking (Supplementary Fig. 7, Supplementary Data 1). The crosslinking data match the structural data very well (Supplementary Fig. 7c), and unambiguously localize XPG. The N-terminal region of XPG specifically crosslinks to the FeS and Arch domains in XPD, including the plug element, whereas the C-terminal extension of XPG crosslinks mostly to XPB and p52 (Supplementary Fig. 7a, d). In addition, XPG crosslinks to XPD at its binding site for the kinase module (Supplementary Fig. 7d), suggesting that XPG competes with the kinase module for XPD binding and explaining how XPD inhibition by the kinase module is relieved (Fig. 4e). These data suggest that XPG facilitates lesion scanning by blocking the kinase module-binding site on XPD and directly stimulating XPD helicase activity. We note that XPG may bind to TFIIH in alternative ways during other TFIIH functions, in which XPD is not bound to DNA^5–7,33^.

## Discussion

Taken together, our structure–function analysis extends our understanding of the NER pathway (Supplementary Fig. 8). The DNA lesion is first recognized by XPC, which recruits TFIIH^1,13^. XPA and XPG then displace the kinase module, stabilize an alternative conformation of TFIIH, and remove the inhibitory plug from the XPD pore. XPA and XPG also stimulate XPB and XPD, and this may facilitate DNA opening^4,14,18,19^ and XPD migration in the 3′ direction to scan the DNA strand for the lesion^13^. XPA chaperons TFIIH–DNA interactions and anchors the NER machinery to the 5′-edge of the repair bubble, where it is ideally positioned to recruit XPF-ERCC1 and complete the repair assembly when the lesion is encountered^16^. The two endonucleases XPF-ERCC1 and XPG can now incise the lesion-containing DNA strand near the 5′- and the 3′-edge of the repair bubble, respectively, to remove the lesion-containing DNA fragment^1^. We acknowledge that, while our manuscript was in revision, a manuscript became available online^34^ that provides a high-resolution free TFIIH structure and includes a mapping of disease mutations consistent with the one described here.

## Methods

### Cloning and protein expression

Vectors encoding full-length XPA, XPG, XPF, ERCC1, RPA1, RPA2, RPA3, XPB, XPD, p62, p52, p44, p34, MAT1, CDK7, cyclin H, and TTDA were ordered from Harvard Medical School PlasmID Repository and used as a template for gene amplification by PCR. Amplified genes were cloned into MacroBac vectors via ligation-independent cloning, as described^35^. ERCC1, p52, p34, and TTDA were cloned into 438A (Addgene: 55218); XPA, XPF, XPB, p62, p44, MAT1, CDK7, and CycH into 438B (Addgene: 55219); and XPG and XPD into 438C vector (Addgene: 55220), which resulted in no tag, N-terminal 6× His or N-terminal 6× His followed by a maltose-binding protein, respectively. XPF and ERCC1, MAT1, CDK7, and CycH, as well as XPB, p62, p52, p44, p34, and TTDA were combined into a single vector via restriction digestion and ligation-independent cloning^35^. RPA1 was cloned in 11B (Addgene: 48295), and RPA 2 and 3 were cloned into 11A vectors (Addgene: 48294), followed by assembly of all RPA subunits into one vector by successive ligation-independent cloning reactions. All tags were separated from the gene with a tobacco etch virus protease site. All mutant proteins used here (CDK7:D137R, CDK7:T170A, XPG:E791A, XPD:K48R) were produced by round-the-horn side-directed mutagenesis and purified as their wild-type counterparts.

RPA complex was expressed in *Escherichia coli* and purified as described^36^. TFIIH kinase module comprised of MAT1, CDK7, or CDK7 mutants and cyclin H was expressed and purified as described^37^. All other proteins and protein complexes were expressed in insect cells and purified as described below. Bacmid preparation and V0 and V1 virus production was as described previously^38^. Core TFIIH was produced by co-infecting the cells with two V1 viruses: virus encoding XPB, p62, p52, p44, p34, and TTDA and a virus encoding XPD or XPD mutant. All proteins were expressed in Hi5 cells grown in ESF-921 media (Expression Systems, Davis, CA, USA) at 27 °C. Typically, 600 ml culture was infected with 500 μl of V1 virus and grown for 48–72 h prior harvesting by centrifugation (30 min, 4 °C, 500 × *g*). Cell pellet was resuspended in lysis buffer; 400 mM KCl, 20% glycerol (v/v), 20 mM KOH-HEPES pH 7.0, 5 mM β-mercaptoethanol, 30 mM imidazole pH 8.0, 0.284 μg ml^−1^ leupeptin, 1.37 μg ml^−1^ pepstatin A, 0.17 mg ml^−1^ phenylmethanesulfonylfluoride (PMSF), and 0.33 mg ml^−1^ benzamidine for the core TFIIH and 400 mM NaCl, 20 mM Tris-HCl pH 7.9, 10% glycerol (v/v), 1 mM dithiothreitol (DTT), 30 mM imidazole pH 8.0, 0.284 μg ml^−1^ leupeptin, 1.37 μg ml^−1^ pepstatin A, 0.17 mg ml^−1^ PMSF, and 0.33 mg ml^−1^ benzamidine for other proteins. Cell suspension was flash frozen in liquid nitrogen and stored at −80 °C.

### Protein purification

All purification steps were performed at 4 °C and all buffers were filtered and thoroughly degassed immediately before use. Cells were thawed in a water bath operating at 30 °C and opened by sonication. The lysate was clarified by centrifugation (18,000 × *g*, 30 min), followed by ultracentrifugation (235,000 × *g*, 60 min). In case of core TFIIH, the clarified lysate was first filtered using 0.8-µm syringe filters (Millipore) and loaded onto HisTrap HP 5 ml (GE Healthcare, Little Chalfont, UK). The column was washed with 10 column volumes (CV) of lysis buffer, followed by 20 CV of high salt wash (800 mM KCl, 20% glycerol (v/v), 20 mM KOH-HEPES pH 7.0, 5 mM β-mercaptoethanol, 30 mM imidazole pH 8.0, 0.284 μg ml^−1^ leupeptin, 1.37 μg ml^−1^ pepstatin A, 0.17 mg ml^−1^ PMSF, and 0.33 mg ml^−1^ benzamidine). Column was washed again with 5 CV of lysis buffer and protein was subsequently eluted with a gradient of 0–80% elution buffer (400 mM KCl, 20% glycerol (v/v), 20 mM KOH-HEPES pH 7.0, 5 mM β-mercaptoethanol, 500 mM imidazole pH 8.0, 0.284 μg ml^−1^ leupeptin, 1.37 μg ml^−1^ pepstatin A, 0.17 mg ml^−1^ PMSF, and 0.33 mg ml^−1^ benzamidine). Fractions were checked on NuPAGE 4–12% Bis–Tris Protein Gels (Invitrogen) for the presence of all core TFIIH subunits, and appropriate fractions were pulled and mixed with 10 ml of amylose resin (New England BioLabs) pre-equilibrated in washing buffer (400 mM KCl, 20% glycerol (v/v), 20 mM KOH-HEPES pH 7.0, 5 mM β-mercaptoethanol, 2 mM MgCl_2_, and 10 μM ZnCl_2_). The protein solution was incubated with the beads for 1 h while rotating. The amylose resin was poured into Econo-Pac Chromatography columns (BioRad) and washed with 5 CV of washing buffer. Protein was eluted with washing buffer containing 100 mM maltose. Protein-containing fractions were pooled, mixed with 2 mg of TEV protease, and dialyzed against 2 l of dialysis buffer overnight (250 mM KCl, 20% glycerol (v/v), 20 mM KOH-HEPES pH 7.0, 5 mM β-mercaptoethanol, 2 mM MgCl_2_, and 10 μM ZnCl_2_). The dialyzed sample was applied to DEAE (GE Healthcare) and heparin column (GE Healthcare) in tandem and washed with 20 CV of dialysis buffer. After the removal of DEAE column, protein was eluted with a gradient of elution buffer 0–100% (1 M KCl, 20% glycerol (v/v), 20 mM KOH-HEPES pH 7.0, 5 mM β-mercaptoethanol, 2 mM MgCl_2_, and 10 μM ZnCl_2_). Peak fractions were pooled, concentrated with Amicon Millipore 15 ml 100,000 MWCO centrifugal concentrator, and applied to Superose 6 increase 10/300 GL column (GE Healthcare) equilibrated in storage buffer (400 mM KCl, 20% glycerol (v/v), 20 mM KOH-HEPES pH 7.0, 5 mM β-mercaptoethanol, 2 mM MgCl_2_). Peak fractions were again concentrated, aliquoted, flash frozen, and stored at −80 °C.

XPF-ERCC1-, XPG-, and XPA-containing lysate was applied onto GE HisTrap HP 5 ml (GE Healthcare, Little Chalfont, UK) equilibrated in lysis buffer (in case of XPA, all downstream steps were performed in the presence of 5 mM β-mercaptoethanol and 10 μM ZnCl_2_ instead of 1 mM DTT). The column was washed with 20 CV of high salt buffer (800 mM NaCl, 20 mM Tris-HCl pH 7.9, 10% glycerol (v/v), 1 mM DTT, 30 mM imidazole pH 8.0, 0.284 μg ml^−1^ leupeptin, 1.37 μg ml^−1^ pepstatin A, 0.17 mg ml^−1^ PMSF, and 0.33 mg ml^−1^ benzamidine). After 5 CV wash with the lysis buffer, proteins were eluted with the elution buffer gradient 0–80% (400 mM NaCl, 20 mM Tris-HCl pH 7.9, 10% glycerol (v/v), 1 mM DTT, 500 mM imidazole pH 8.0, 0.284 μg ml^−1^ leupeptin, 1.37 μg ml^−1^ pepstatin A, 0.17 mg ml^−1^ PMSF, and 0.33 mg ml^−1^ benzamidine). Pulled peak fractions were processed differently for different proteins. XPF-ERCC1- and XPA-containing protein solutions were directly mixed with 2 mg of TEV protease and dialyzed against 1 liter of dialysis buffer (400 mM NaCl, 20 mM Tris-HCl pH 7.9, 10% glycerol (v/v), 1 mM DTT). XPG solution was mixed with 10 ml of amylose resin (New England BioLabs) pre-equilibrated in dialysis buffer. Solution was mixed for 1 h and subsequently poured into Econo-Pac Chromatography columns (BioRad). Column was washed with 10 CV of dialysis buffer followed by elution with dialysis buffer containing 100 mM maltose. Protein-containing fractions were pulled, mixed with 2 mg of TEV protease, and dialyzed against 1 liter of dialysis buffer. Dialyzed solutions containing XPF:ERCC1, XPA, and XPG were loaded on GE HisTrap HP 5 ml and flow through fractions were collected. Protein-containing fractions were checked for contaminants on NuPAGE 4–12% Bis–Tris Protein Gels (Invitrogen), pulled, concentrated with appropriate Amicon Millipore 15 ml centrifugal concentrator, and applied onto Superdex 75 10/300 equilibrated in storage buffer (400 mM NaCl, 20 mM NaOH:HEPES pH 7.5, 10% glycerol (v/v), 10 μM ZnCl_2_, and 5 mM β-mercaptoethanol) for XPA and Superdex 200 10/300 increase (GE Healthcare) equilibrated in a different storage buffer (400 mM NaCl, 20 mM NaOH:HEPES pH 7.5, 10% glycerol (v/v), 1 mM DTT) for the rest of the proteins. Peak fractions were pooled, concentrated, aliquoted, flash frozen, and stored at −80 °C.

### Helicase and translocase assays

H1 and H2 DNA sequences (Supplementary Table 1) were used for monitoring the helicase activity in 5′–3′ direction and H3 and H4 for monitoring the helicase activity in 3′–5′ direction. DNA annealing reaction contained fluorescent DNA primer (25 μM) and quenching DNA oligo (37.5 μM) dissolved in water. Annealing was performed in a thermocycler by heating up the DNA solution to 95 °C for 5 min, followed by slow cooling (1 °C/min) to 4 °C. Typical unwinding reactions of 20 μl final volume contained 0.4 pmol of DNA duplex and 8 pmol of core TFIIH in 100 mM KCl, 20 mM KOH:HEPES pH 7.0, 5% glycerol, 0.2 mg ml^−1^ bovine serum albumin, 3 mM phosphoenolpyruvate, 10 mM MgCl_2_, 1 mM DTT, and excess amount of pyruvate kinase (Sigma). When the effect of DNA repair factors on unwinding was measured, we supplemented the reaction with 24 pmol of the corresponding factor. The reaction mixture was preincubated at 26 °C for 10 min. The reaction was started by addition of ATP (2 mM final) and the unwinding was monitored at 26 °C by using the Infinite M1000Pro reader with excitation wavelength 495 nm, emission wavelength 520 nm, and gain of 150. Percentage of unwound product was calculated by dividing the observed fluorescence intensity by the intensity of the fluorescent primer in the reaction buffer (mimicking fully unwound DNA).

DNA unwinding monitored by stopped-flow was performed in the same buffer conditions and with the same final protein and DNA concentrations as above. The core TFIIH preincubated with XPA or XPG was rapidly mixed with equal volume of ATP (2 mM final) in the SX-20MV stopped-flow apparatus (Applied Photophysics). FAM fluorescence was monitored upon excitation at 465 nm after passing through KV500 cut-off filter (Schott). All time courses shown represent average of five technical replicates. Initial rate of DNA unwinding was calculated using Prism (Graphpad software) by fitting the initial linear part of the fluorescence trace.

Triplex displacement assay was performed in a similar way as previously described^11^. Ten-microliter annealing reaction for triplex displacement assay contained T1 (30 μM) and T2 (25 μM) oligo (Supplementary Table 1) in 25 mM MES pH 5.5 and 10 mM MgCl_2_. The reaction was heated to 95 °C for 5 min followed by slow cooling (1 °C/min) to 4 °C. After cooling, the reaction was supplemented with 1 μl of florescent T3 oligo (9 μM final), heated to 57 °C, and cooled down to 20 °C at the speed of 1 °C/min. Translocation reactions were preformed exactly as described for the helicase assay, only with triplex DNA as a substrate. A higher core TFIIH input (75 pmol in 20 μl reactions) was used when no stimulatory factors were added (Supplementary Fig. 1c) to obtain a more robust fluorescent signal.

### Kinase activity assay

We used the kinase activity assay to assess the activity of kinase module variants containing CDK7:D137R or CDK7:T170A mutants^39^. As CDK7 phosphorylates the C-terminal domain of the largest RNA polymerase II subunit during transcription initiation, we used purified yeast RNA polymerase II^40^ dephosphorylated with lambda phosphatase during purification^37^ as a substrate in the assay. RNA polymerase II (50 nM final) was mixed with increasing concentrations of kinase module variants (30, 100, 220, and 500 nM final) in final buffer conditions containing 100 mM KCl, 20 mM KOH:HEPES pH 7.5, 3 mM MgCl_2_, 5% glycerol, and 5 mM β-mercaptoethanol and preincubated for 2 min at 30 °C. Reactions were started by the addition of ATP (0.5 mM final) and quenched after 2 min at 30 °C with EDTA (100 mM) and 4× LDS buffer (Invitrogen). Reactions were run on 4–12% Bis-Tris gel in MOPS buffer (ThermoFisher Scientific) and transferred to nitrocellulose membranes (GE Healthcare Life Sciences). The membranes were blocked with 5% (w/v) milk in phosphate-buffered saline (PBS) buffer supplemented with 0.1% Tween 20 for 1 h at room temperature. The membranes were treated with primary antibody (3E8, 1:25 dilution) in 0.25% (w/v) milk in PBS supplemented with 0.1% Tween 20 and incubated at room temperature for 1 h. After several rounds of washing with PBS buffer supplemented with 0.1% Tween 20, the membranes were incubated with horseradish peroxidase-conjugated anti-rat secondary antibody (1:5000 dilution, Sigma-Aldrich A9037) in 0.1% (w/v) milk in PBS supplemented with 0.1% Tween 20 and incubated at room temperature for 1 h. Antibodies were detected with SuperSignal West Pico Chemiluminescent Substrate (ThermoFisher) and the membranes were scanned with ChemoCam Advanced Fluorescence imaging system (Intas Science Imaging).

### Mass spectrometric identification of crosslinking sites

Sample for crosslinking was prepared by mixing core TFIIH, XPA, XPG, and bifurcated DNA scaffold (see “Cryo-EM sample preparation and image processing”) in 1:2:2:1.5 molar ratio in a final buffer containing 150 mM KCl, 10% glycerol, 2 mM MgCl_2_, 20 mM KOH:HEPES pH 7.5, and 5 mM β-mercaptoethanol. The reaction was incubated for 20 min at room temperature before applying to the Superose 6 increase 3.2/300 column equilibrated in final buffer used for the complex formation. Fractions were analyzed by sodium dodecyl sulfate-polyacrylamide gel electrophoresis and Coomassie staining. For crosslinking, purified complexes (~2.2 μM final) were supplemented with BS3 crosslinker (2 mM final, ThermoFisher Scientific) and incubated for 30 min at 30 °C. Reaction was quenched with ammonium bicarbonate (200 mM final) and further incubated for 10 min at 30 °C.

Crosslinked proteins were reduced with 10 mM DTT for 30 min at 37 °C, followed by alkylation with 40 mM iodoacetamide for 30 min at 25 °C. The proteins were digested overnight at 37 °C in the presence of 1 M urea with trypsin in 1:20 (w/w) enzyme-to-protein ratio. The samples were acidified with formic acid (FA) to 0.1% (v/v) final concentration and acetonitrile (ACN) was added to 5% (v/v) final concentration. Peptides were bound to Sep-Pak C18 50 mg sorbent cartridge (Waters), washed with 5% ACN and 0.1% FA (v/v), eluted with 80% ACN and 0.1%FA (v/v), dried under vacuum, and resuspended in 30 µl 30% ACN and 0.1% trifluoroacetic acid (TFA) (v/v). The sample was fractionated on Superdex Peptide PC3.2/30 column (GE Healthcare) at a flow rate of 50 µl min^−1^ of 30% ACN, 0.1% TFA (v/v) and 100 µl fractions corresponding to elution volume 0.9–2.2 ml were collected, dried under vacuum, and resuspended in 20 µl 2% ACN and 0.05% TFA (v/v).

Crosslinked peptides were analyzed on Orbitrap Fusion Tribrid Mass Spectrometer (Thermo Scientific), coupled to Dionex UltiMate 3000 UHPLC system (Thermo Scientific) equipped with an in-house-packed C18 column (ReproSil-Pur 120 C18-AQ, 1.9 µm pore size, 75 µm inner diameter, 30 cm length, Dr. Maisch GmbH). Samples were analyzed as 5 µl injections, separated on 118 min gradient: mobile phase A—0.1% FA (v/v); mobile phase B—80% ACN, 0.08% FA (v/v). The gradient started at 8% B and increased to 48% B in 106 min, then keeping B constant at 90% for 6 min, followed by re-equilibration of the column with 5% B. The flow rate was set to 300 nl min^−1^. MS1 spectra were acquired with the following settings: resolution—120,000; mass analyzer—Orbitrap; mass range—380–1500 *m*/*z*; injection time—60 ms; automatic gain control target—4 × 105. Dynamic exclusion was set to 9 s. For samples of elution volume up to 1.7 ml, only charges 3–8 were included. For subsequent samples, charges 2–8 were included. MS2 spectra settings: resolution—30,000; mass analyzer—Orbitrap; injection time—128 ms; automatic gain control target—5 × 104; isolation window—1.6 *m*/*z*. Fragmentation was enforced by higher-energy collisional dissociation and varied for the three injections at 30%, 28%, and stepped collision energy with step +/− 2% at 28%.

Raw files were converted to mgf format with ProteomeDiscoverer 2.1.0.81 (Thermo Scientific): signal-to-noise ratio—1.5; precursor mass—350–7000 Da. For identification of crosslinked peptides, files were analyzed by pLink (v. 1.23) (pFind group)^41^ with the following settings : crosslinker—BS3 (default settings—lysine and the protein N-terminus); digestion enzyme—trypsin; missed cleavage sites—2; fixed modification—carbamidomethylation of cysteines; variable modification—oxidation of methionines; precursor mass tolerance filtering—10 ppm; fragment ion mass tolerance—20 ppm; false discovery rate—1% (Supplementary Data 1). The sequence database contained all proteins within the complex. Crosslinking figures were created with XiNet^42^ and Xlink Analyzer plugin in Chimera^43^.

### Cryo-EM sample preparation and image processing

Sample was prepared by mixing pre-annealed DNA scaffold (5′-CAAAGTCACGACCTAGACACTGCGAGCTCGAATTCACTGGAGTGACCTC-3′; 5′-GAGGTCACTCCAGTGAATTCGAGCTCGCAACAATGAGCACATACCTAGT-3′) with core TFIIH, XPA and XPG:E791A in 1.5:1:3:3 molar ratio in final buffer containing 150 mM KCl, 20 mM KOH:HEPES pH 7.0, 10% glycerol, 2 mM MgCl_2_, and 5 mM β-mercaptoethanol. XPG:E791A endonuclease mutant was used to prevent DNA cleavage during the sample preparation^44^. The sample mixture was applied to a sucrose gradient in order to purify the complex from excess factors and fix it with glutaraldehyde^45^. The sucrose gradient was prepared with BioComp Gradient Master 108 (BioComp Instruments) by mixing equal volume of heavy (30% (w/v) sucrose, 150 mM KCl, 2 mM MgCl_2_, 5 mM β-mercaptoethanol, 20 mM KOH-HEPES pH 7.5, and 0.1% glutaraldehyde) and light solutions (10% (w/v) sucrose, 150 mM KCl, 2 mM MgCl_2_, 5 mM β-mercaptoethanol, and 20 mM KOH-HEPES pH 7.5) in 5 ml centrifugation tubes. After 16 h of centrifugation at 4 °C and 175,000 × *g*, the gradient was fractionated and glutaraldehyde was quenched with lysine (50 mM final) and aspartate (20 mM final). Fractions were dialyzed in Slide-A-Lyzer MINI Dialysis Devices (2 ml and 20 kDa cut-off) (ThermoFisher Scientific) for 10 h against buffer containing 100 mM KCl, 20 mM KOH-HEPES pH 7.5, 1 mM MgCl_2_, 1 mM DTT, 0.5% glycerol (v/v), and 0.004% n-octyl glucoside (w/v). Dialyzed samples were immediately used for cryo-grid preparation. Four microliters of sample was applied to glow-discharged R2/2 gold grids (Quantifoil), which were blotted for 5 s and plunge-frozen in liquid ethane with a Vitrobot Mark IV (FEI) operated at 4 °C and 100% humidity.

Micrographs of the sample were acquired on a FEI Titan Krios G2 transmission electron microscope with a K2 summit direct electron detector (Gatan). Data acquisition was automated with the FEI EPU software package. Micrographs were acquired at a nominal magnification of ×130,000 (1.05 Å/pixel) using a dose rate of 4.55 e^−^/Å^2^ per s over the time of 9 s that resulted in a total dose of 41 e^−^/Å^2^ fractionated over 40 frames. CTF correction, motion correction, and particle picking was done on-the-fly using Warp^46^. Automated picking in retrained BoxNet implemented in Warp^46^ yielded a total of 1,354,997 particles from 8993 micrographs, which were further subjected to two-dimensional (2D) classification in CryoSPARC^47^. After 2D cleaning, 950,000 particles were used for heterogeneous refinement in CryoSPARC. Three ab initio classes obtained from the first 300,000 particles picked during data acquisition were used as an input for the refinement. The class showing clear core TFIIH features was further three-dimensional (3D) classified into six classes using RELION-3^48^. Particles corresponding to the best 3D class were subjected to CTF refinement and Bayesian polishing. Particles were 3D refined and post-processed with automatic *B*-factor determination in RELION. Final map showed an overall resolution of 3.6 Å according to the gold-standard Fourier shell correlation 0.143 criterion. Owing to flexibility of peripheral regions of core TFIIH, we improved the map quality for five different regions of the complex by focused 3D classification and refinement (processing tree is depicted in Supplementary Fig. 2d). The classifications were performed with particles contributing to the final map without image alignment to speed up the calculations. Masks encompassing the regions of interest were created with UCSF Chimera^49^ and RELION. 3D classification of the DNA duplex revealed two alternative DNA conformations within the complex (Supplementary Fig. 2d).

### Model building

The final cryo-EM map and focused refined maps were used for model building. The final map was denoised in Warp 1.0.6^46^. Structures of ATPase lobes 1 and 2 of XPB, XPD, p44 vWA-like domain, and p52 C-terminus (residues 383–458) from the TFIIH structure (PDB code 5OF4 [10.2210/pdb5OF4/pdb])^6^, as well as the crystal structure of p34 vWA-like domain bound to p44 RING domain (PDB code 5O85 [10.2210/pdb5O85/pdb])^50^ were rigid-body fitted into our cryo-EM density in UCSF Chimera^49^ and manually adjusted in COOT^51^. Owing to high quality of the EM density, the NTE domain and part of the DRD domain (residues 71–199 and 266–300), as well as the p52 region that interacts with XPB (residues 307–382) were built de novo guided by secondary structure prediction in PSIPRED^52^ and bulky amino acid side chains as sequence registers. In case of XPD, we did not observe EM density corresponding to residues 273–325, so we removed this part of the structure. We observed a very strong density for the iron-sulfur cluster indicating that the ligand was not damaged or dissociated during protein expression and purification, as well as sample preparation for cryo-EM. The N-terminal region of p52 (residues 18–289) and zinc-fingers belonging to subunits p34 and p44 were modeled with SWISS-Model^53,54^ based on the yeast p52 counterpart (PDB code 5OQJ [10.2210/pdb5OQJ/pdb])^7^ and manually adjusted in COOT. Interestingly, the p34 zinc finger region in human contains additional cysteine (C257) and histidine (H258) residues not present in the yeast counterpart that allows binding of an additional zinc ion. The smallest TFIIH subunit TTDA (p8) was generated in Modeller^55^ with the yeast TTDA structure as a reference (PDB code 5OQJ [10.2210/pdb5OQJ/pdb])^7^, rigid-body fitted in our density using UCSF Chimera, and manually adjusted in COOT. The nuclear magnetic resonance structure of truncated human XPA (PDB code 1XPA [10.2210/pdb1XPA/pdb])^56^ was also docked in our density as a rigid body and adjusted in COOT. We observed additional helical density that extends from the C-terminus of the docked structure toward the ATPase lobe 2 of XPB when the map is filtered to lower resolution. Secondary structure prediction with PSIPRED shows that residues following the docked XPA C-terminus form a helix, so we extended the C-terminal helix in COOT guided by the cryo-EM density (Supplementary Fig. 3h).

DNA sequence was assigned based on the position of the DNA duplex–single strand junction; however, protein binding to the junction could induce additional DNA melting so register shifts cannot be excluded. DNA duplex was built by docking ideal B-DNA into the density, followed by manual adjustments in COOT. Several rounds of real space refinement and geometry optimization with secondary structure restraints (including base pairing and base stacking restraints) were performed in PHENIX^57^. The DNA duplex–single strand junction and single-strand extensions were manually built in COOT. The EM density for the 5′–3′ DNA single strand showed clear separation of sugar, phosphate, and DNA bases for nucleotides A30–G35 and for C40–A41. The decreased quality of EM map for nucleotides A36–A39 and T42, presumably due to increased flexibility of DNA between XPD helicase lobes, allowed the trajectory of DNA to be determined, but the nucleotides were positioned manually in COOT guided by the structure of NS3 helicase in complex with DNA^58^ and real space refined in PHENIX. All core TFIIH subunits, XPA, and DNA were first real-space refined in PHENIX separately in their corresponding focused classified maps. Then all components were combined and real-space refined together in the global map. The final model was validated using Molprobity^59^ (Supplementary Table 2). Figures were generated using PyMOL (Schrödinger LLC) and UCSF Chimera^49^.

### Reporting summary

Further information on research design is available in the Nature Research Reporting Summary linked to this article.

## Supplementary information

Supplementary Information

Description of Additional Supplementary Files

Supplementary Movie 1

Supplementary Data 1

Reporting Summary

Source Data

## Data Availability

The cryo-EM density reconstructions were deposited with the Electron Microscopy Data Base (code EMD-4970). The main cryo-EM map is composed of focused refined maps that were deposited under the same accession code together with the globally refined map and all corresponding half maps. The final model was deposited with the Protein Data Bank (code 6RO4). The crosslinking mass spectrometric data have been deposited to the ProteomeXchange Consortium via the PRIDE^60^ partner repository with the dataset identifier PXD013947. All other relevant data supporting the key findings of this study are available within the article, its Supplementary Information, and Source Data or from the corresponding author upon reasonable request.
