# Supplementary material for: Structural basis of TFIIH activation for nucleotide excision repair
Source: Nat Commun. 2019 Jun 28;10:2885. doi: 10.1038/s41467-019-10745-5 (PMC6599211; doi:10.1038/s41467-019-10745-5)
Supplement: Supplementary file 1 — Supplementary Information [file 41467_2019_10745_MOESM1_ESM.pdf]

Supplementary Information for:

Structural basis of TFIIH activation for nucleotide excision repair

Kokic et al.

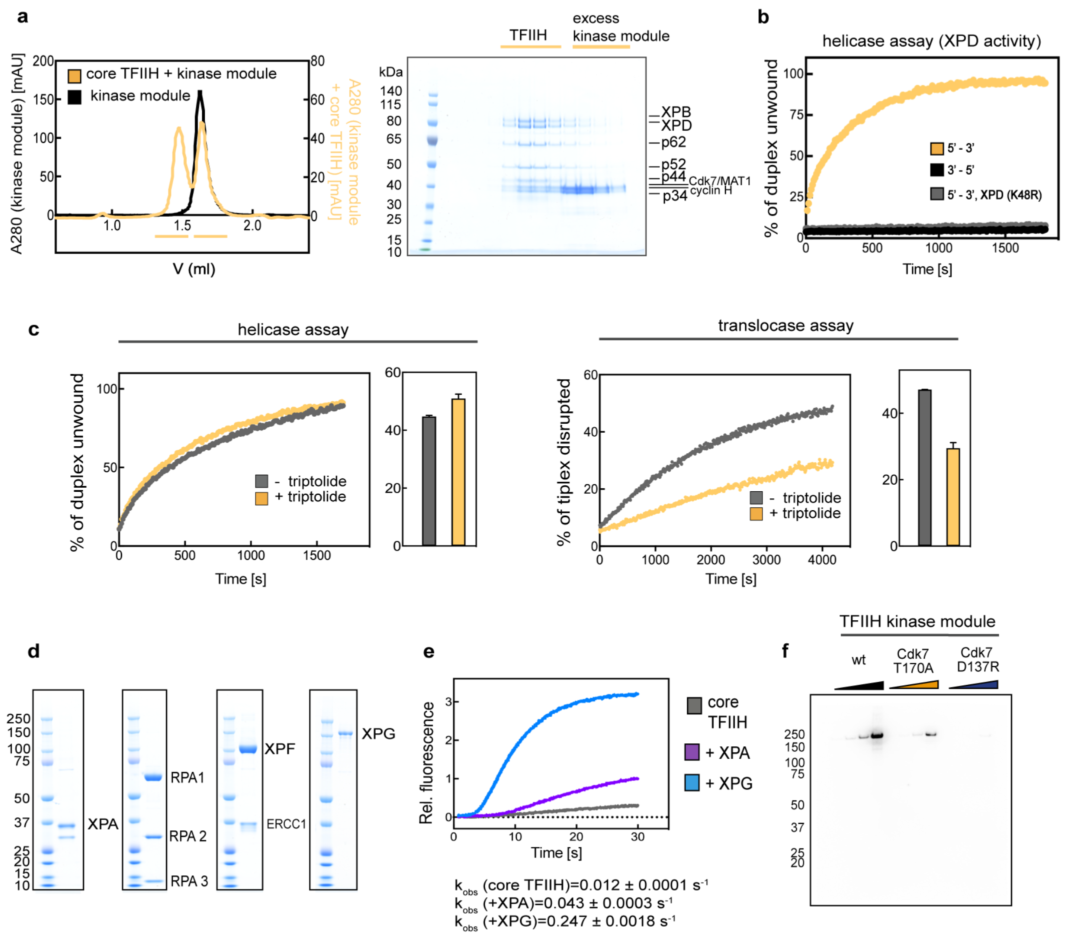

Supplementary Figure 1 | Biochemical characterization of TFIIH ATPases

a. Reconstitution of the TFIIH complex by size-exclusion chromatography. Chromatograms show elution profile for kinase module in black and the mixture of the core TFIIH and the kinase module in yellow. Two peaks detected for the mixture of the core TFIIH and the kinase (yellow lines) were resolved by SDS-PAGE. First peak contains reconstituted TFIIH and the second peak contains the excess kinase module. Gel also demonstrates the purity of TFIIH preparations. Source Data are provided in the Source Data file.

b. Polarity of XPD helicase. DNA unwinding was monitored by a fluorescence resonance energy transfer - based assay. Fluorescence traces of the core TFIIH unwinding in the 5' – 3' and the 3' – 5' direction are shown in yellow and black, respectively. DNA unwinding of the core TFIIH containing XPD K48R mutant is shown in gray. Shown traces are representative of 3 independent experiments.

c. DNA translocation by the core TFIIH is mediated by XPB. DNA unwinding and triplex disruption were monitored by a fluorescence resonance energy transfer - based assay. Helicase (left) and translocase (right) activities of the core TFIIH in the presence (yellow) or absence (gray) of triptolide (100 µM final) are shown. Triptolide specifically inhibits the XPB enzyme[1] and affects the core TFIIH translocation, without affecting the XPD helicase activity, indicating that XPB mediates DNA translocation. Bar graphs show the percentage of unwound duplex after 300 s (left) and the percentage of disrupted triplex after 4000 s (right) (*n*=2, ± SD). Source Data are provided in the Source Data file.

d. Purified NER factors resolved by SDS-PAGE and visualized by Coomassie staining.

e. Stopped-flow measurement of DNA unwinding. TFIIH core (gray) was pre-incubated with excess XPA (purple) or XPG (blue) and rapidly mixed with ATP in a stopped-flow apparatus. DNA unwinding was monitored by a fluorescence resonance energy transfer - based assay. Fluorescence traces represent the average of 5 measurements. Initial linear parts of the traces were fitted with Prism software to obtain the initial rate of DNA unwinding, as indicated below the graph.

f. Activity of kinase module variants. Different kinase module variants were incubated with the dephosphorylated yeast RNA-polymerase II and the phosphorylation status of the C-terminal domain of Rpb1 was probed by Western blotting. CDK7:T170A exhibits a much weaker kinase activity compared to the wild type CDK7, while CDK7:D137R mutant is kinase dead. Source Data are provided in the Source Data file.

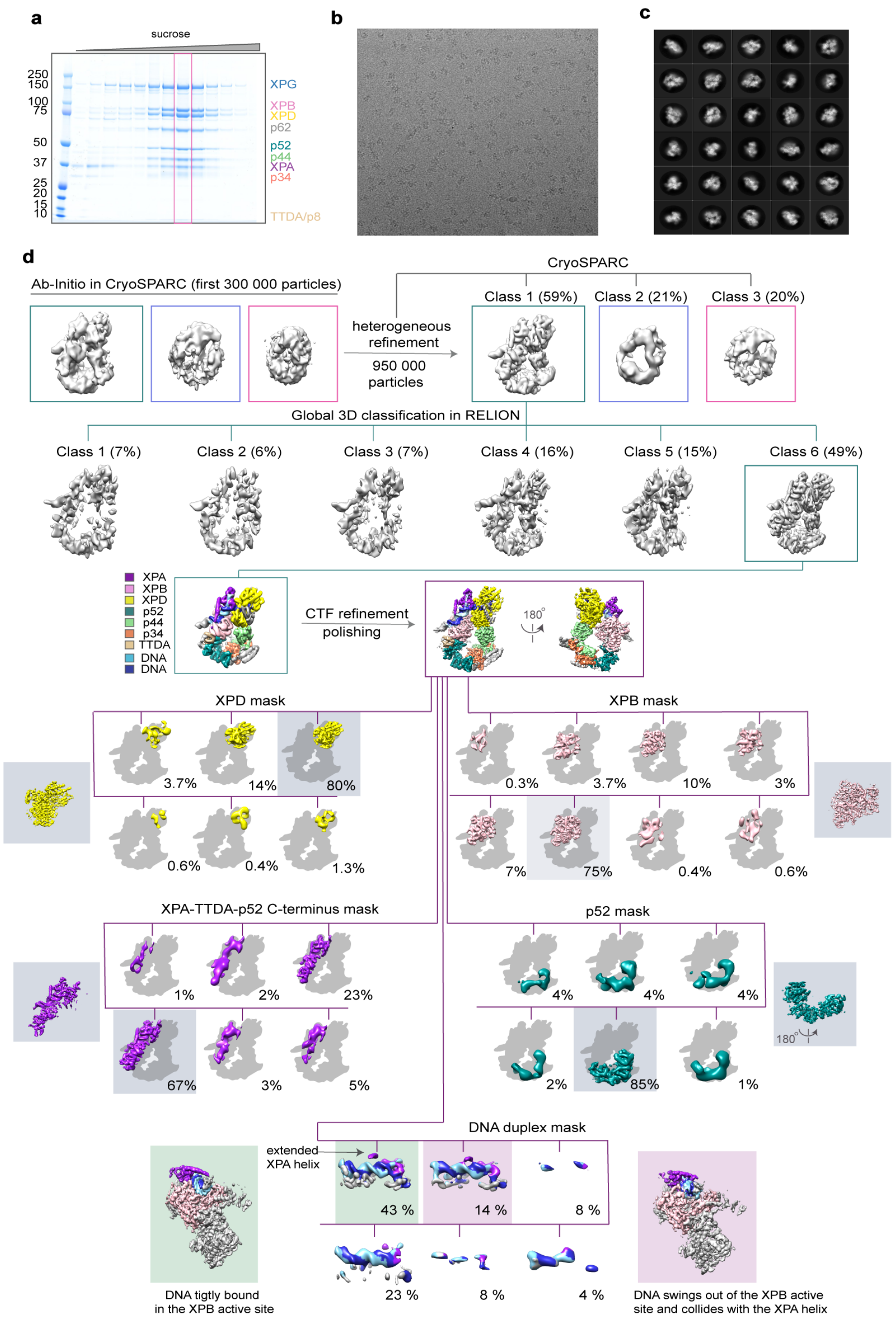

Supplementary Figure 2 | Cryo-EM data processing

a. Core TFIIH-XPA-XPG-DNA complex formation for cryo-EM. Sucrose gradient fractions from the control without glutaraldehyde were analyzed by SDS-PAGE and Coomassie staining. Framed fraction in the presence of glutaraldehyde was used for structure determination. Source Data are provided in the Source Data file.

b. Representative micrograph shows nicely spaced individual particles.

c. Thirty representative 2D classes obtained after the final 3D classification.

d. Processing tree for structure determination of the core TFIIH-XPA-DNA complex. Particle class-distribution after 3D classification is indicated next to the corresponding map. The final map obtained by global refinement is color coded by subunits as indicated. 5 focused classifications used to improve different parts of the structure are shown over the gray TFIIH silhouette for context. Classes used for the refinement are indicated with colored background and the refined structure is shown next to the corresponding 3D classes.

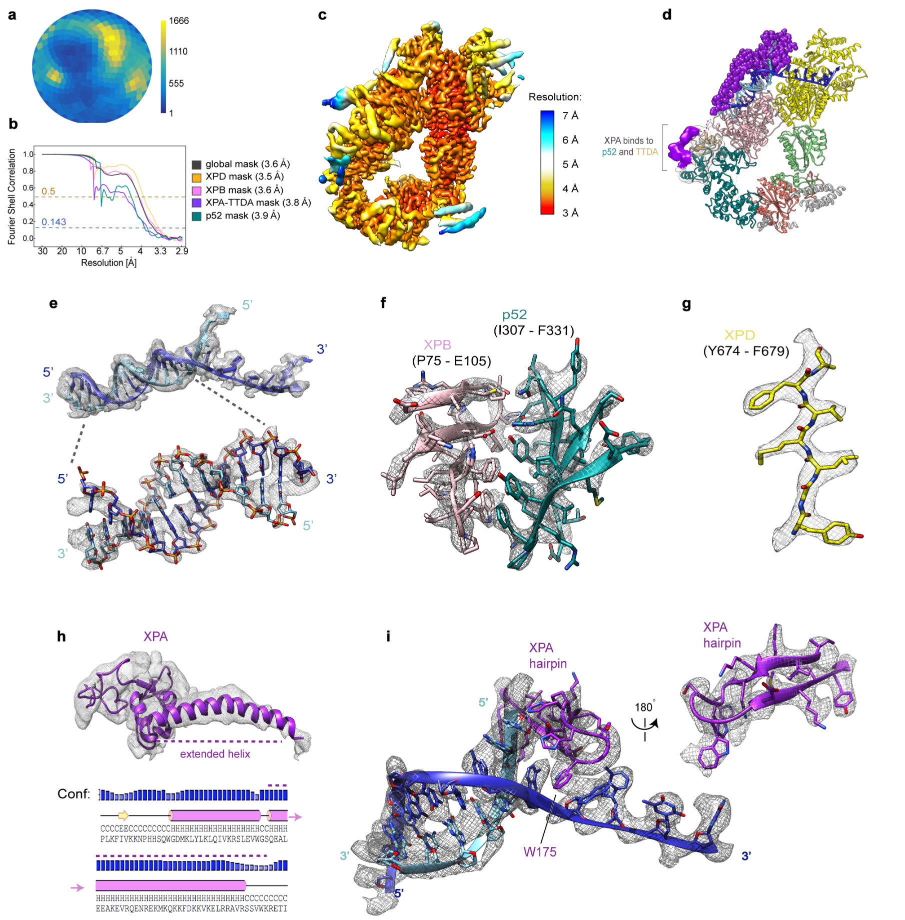

Supplementary Figure 3 | Cryo-EM map quality

a. Angular distribution plot for particles contributing to the final reconstruction. Number of particles assigned to a particular orientation is color-coded as indicated.

b. Fourier shell correlation plots for the final and focused-classified maps.

c. Local resolution estimates for the final map.

d-i. Examples of the final map quality. d. Additional XPA density (purple) contacting p52 dimerization domain and TTDA/p8 is observed at lower resolution and it was not used for model building. XPA is shown as purple spheres and TFIIH subunits are color coded as in Fig. 2a. e. Cryo-EM density corresponding to the bifurcated DNA scaffold. Close-up view of the double stranded DNA region shows a clear separation of the density corresponding to phosphates, sugar and DNA bases. f. De novo built interface between the XPB and p52 subunits. Bulky side chains were used to assign the protein register. g. Individual beta-strand from the second helicase lobe of XPD. The high map quality allows the protein backbone tracing and the placement of side-chains. h. Cryo-EM density for the extended XPA helix directs the placement of the secondary structure element in the lower resolution map. PSIPRED tool[2] predicts the formation of the long helix with high confidence. i. Cryo-EM density for the XPA intercalating hairpin. W175 is indicated.

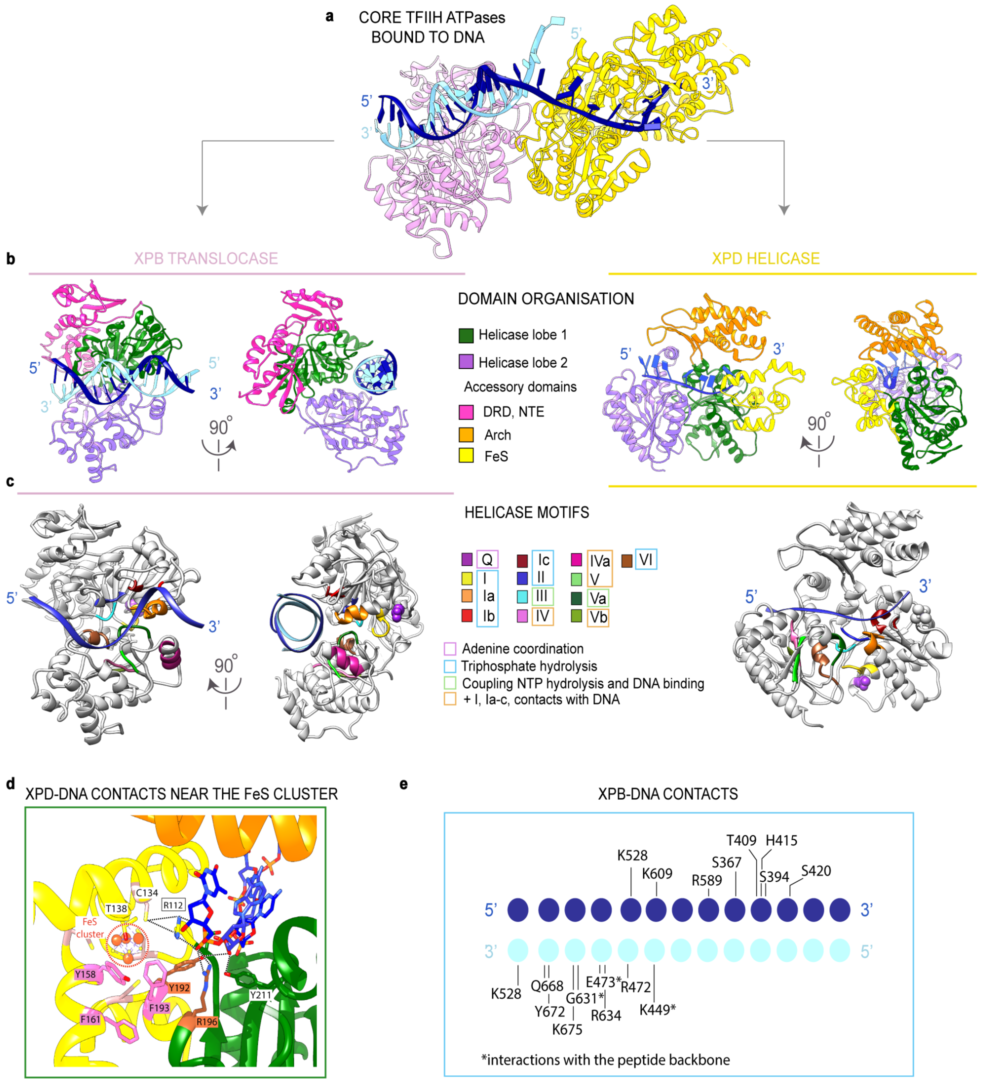

Supplementary Figure 4 | Detailed analysis of XPB and XPD helicase structures

a. Ribbon representation of XPB (pink) and XPD (yellow) bound to the DNA.

b. Domain organization of XPB (left) and XPD (right). Helicase lobes and accessory domains are color coded as indicated.

c. Helicase motifs colored for XPB (left) and XPD (right) according to[3]. In XPB structure it is clearly visible that the 5'-3' DNA strand interacts with the helicase motifs.

d. XPD-DNA interactions near the FeS cluster. The FeS domain is shown in yellow, the Arch domain in orange and the ATPase lobe 1 in green. Residues implicated in lesion scanning are highlighted in brown and aromatic residues surrounding FeS cluster in pink.

e. Schematic representation of XPB-DNA interactions.

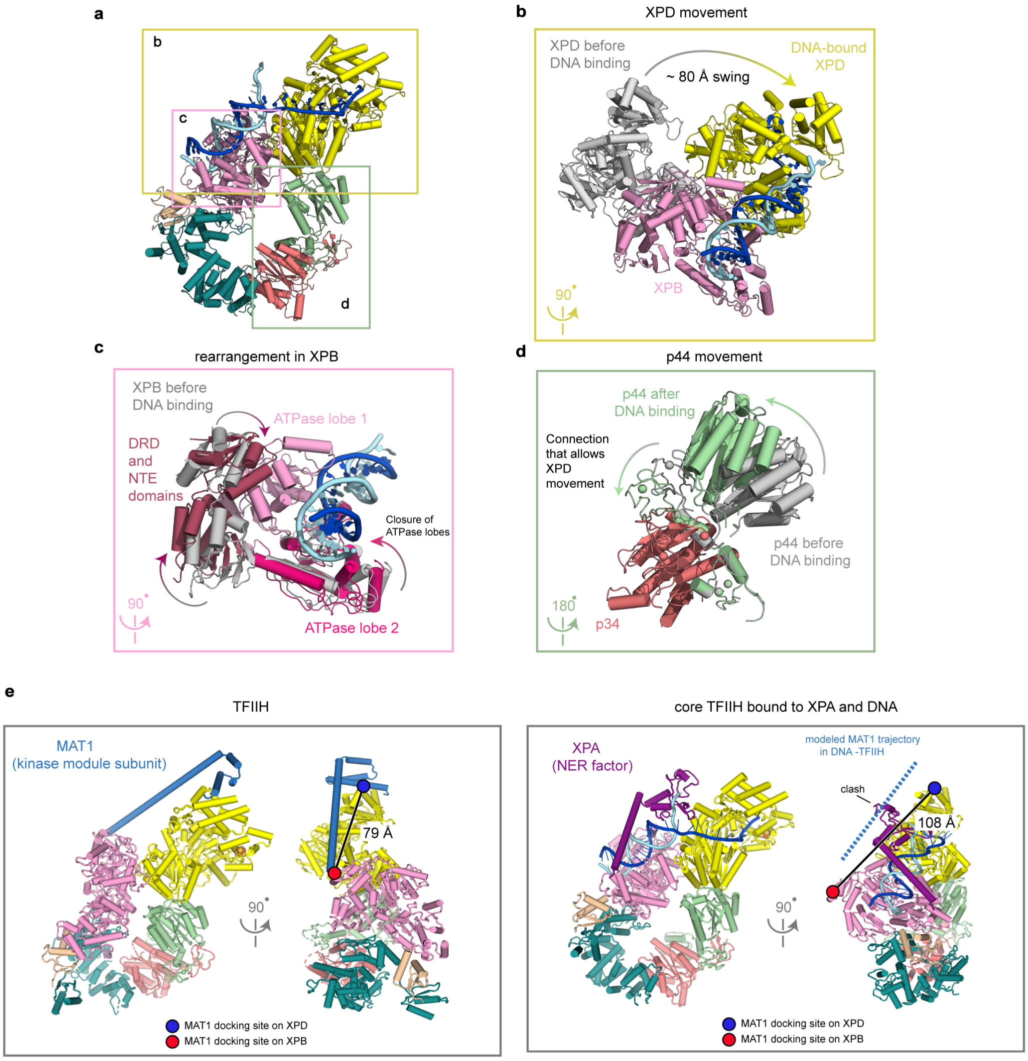

Supplementary Figure 5 | Large-scale structural changes in TFIIH

a. The core TFIIH-DNA structure shown for context and color-coded as in Fig. 2a.

b-d. Superpositions of different TFIIH subunits before and after DNA binding show inter- and intra-subunit flexibility. TFIIH structure in absence of DNA is always shown in gray. The structure was modeled by fitting TFIIH domains built here into the EM density for the kinase-bound TFIIH[4] for easier comparison, followed by a real-space refinement in PHENIX. b. The structures with and without the DNA were aligned on the ATPase lobe 1 of XPB. XPD swings ~80 Å during DNA loading (R272 in corresponding structures was used for the distance measurement). c. Internal rearrangements in XPB. XPB domains are color-coded as follows: NTE and DRD domains in dark pink, ATPase lobe 1 in pink and ATPase lobe 2 in hotpink. Binding to DNA duplex induces closure of XPB ATPase lobes and substantial rearrangement of accessory domains DRD and NTE. d. Flexile interface between p44 and p34 comprised of several Zn-fingers supports the large-scale movement of XPD during DNA loading. **e.** Structural basis of the core TFIIH activation for DNA repair. Kinase-bound core TFIIH (left) and DNA-bound core TFIIH-XPA (right) structures are shown. Front views show that MAT1 and XPA bridge the core TFIIH ATPases in the kinase-bound structure and in the DNA-bound structure, respectively. Side views were aligned on XPD and show how XPB and XPD reorient during DNA and XPA binding. Distance between the docking sites of MAT1 on XPB (red circle, Q190 residue was used for distance measurement) and XPD (blue circle, V365 was used for distance measurement) on both structures is indicated. The distance increases significantly in the core TFIIH conformation stabilized by DNA and XPA which would induce the kinase module rearrangement on the core TFIIH and may facilitate kinase module dissociation *in vivo*[5]. MAT1 helix trajectory that spans between XPB and XPD was modeled in the DNA-bound structure (dotted blue line) and suggests a potential clash with XPA.

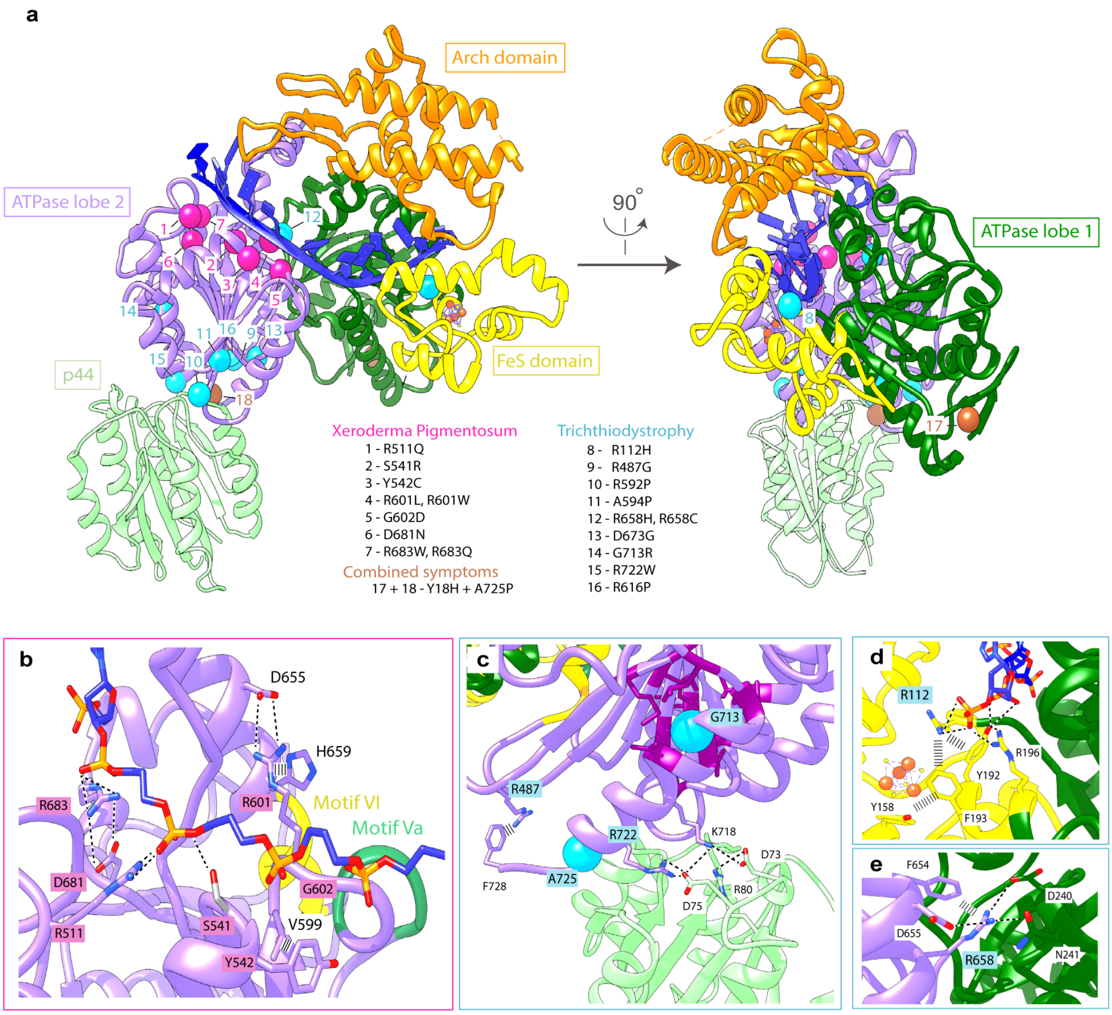

Supplementary Figure 6 | DNA-bound TFIIH core at 3.6Å reveals structural etiology of Xeroderma pigmentosum (XP) and Trichothiodystrophy (TTD)

a. XP and TTD mutations[6-8] mapped onto XPD-DNA structure are shown as pink and blue spheres, respectively. XP mutations cluster around DNA in the ATPase lobe 2 of XPD and TTD mutations cluster at the XPD-p44 interface. A XP-TTD overlap patient carries 2 mutations[9], shown as brown spheres.

b. Zoom-in on XP mutations in the direct vicinity of DNA. Residues mutated in XP are highlighted in pink and their interaction partners in white. Helicase motifs VI and Va are shown in yellow and green, respectively.

c. XPD-p44 interface. A single helix in XPD is affected by four different TTD mutations. Mutated residues are highlighted in blue. G713 and A725 are depicted as blue spheres. The helix carrying TTD mutations interacts with p44 via two salt bridges, R722 (XPD) - D75 (p44), and K718 (XPD) - D73 (p44).

d. R112 residue, mutated in TTD, interacts with the DNA backbone and residues surrounding the iron sulfur cluster.

e. R658 residue, mutated in TTD, engages in the interaction network between the ATPase lobes.

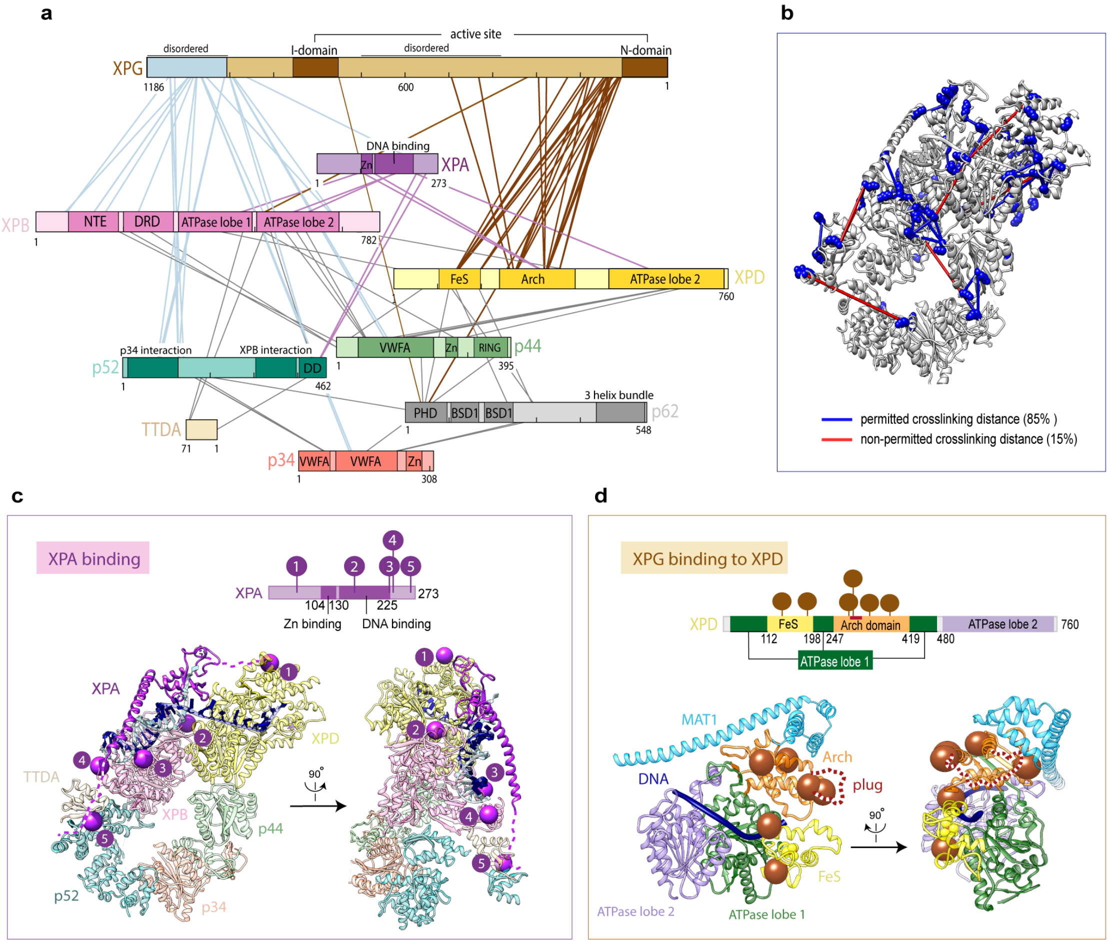

Supplementary Figure 7 | Crosslinking mass-spectrometry network of the core TFIIH-XPA-XPG-DNA complex

a. Crosslinks between XPA and core TFIIH are shown in purple, the disordered XPG C-terminus and core TFIIH in blue, and XPG N-terminal region and core TFIIH in brown. Crosslinks between the core TFIIH subunits are in gray. Only crosslinks with the score above 6 are shown. Crosslinks between the subunits not shown here can be found in the Supplementary Data 1.

b. Validation of crosslinking sites. Crosslinks with the score above 6 were mapped onto core TFIIH-XPA-DNA structure. Colored rods connecting crosslinked residues represent permitted (blue) or non-permitted (red) crosslinking distances, and lysine residues are shown as blue spheres. 85% of mapped crosslink sites fall within the permitted crosslinking distance. Permitted crosslinking distance was set to 30Å and the percentage of mapped crosslink sites within the permitted distance was calculated with Xlink Analyzer plugin in Chimera[10]. 15% of crosslink sites outside the permitted distance likely reflect structural flexibility in solution or arise from technical errors.

c. XPA crosslinks mapped onto core TFIIH-XPA-DNA structure. XPA crosslinks are shown as purple spheres. Schematic representation of XPA with mapped crosslinks is shown above the structure.

d. XPG crosslinks mapped onto XPD-DNA structure. XPD domains are color coded as in Fig. 4, MAT1 subunit of the kinase module is shown in blue and was modeled based on the previous TFIIH structure[4]. XPG crosslinks are shown as brown spheres. Schematic representation of XPD with mapped crosslinks is shown above the structure.

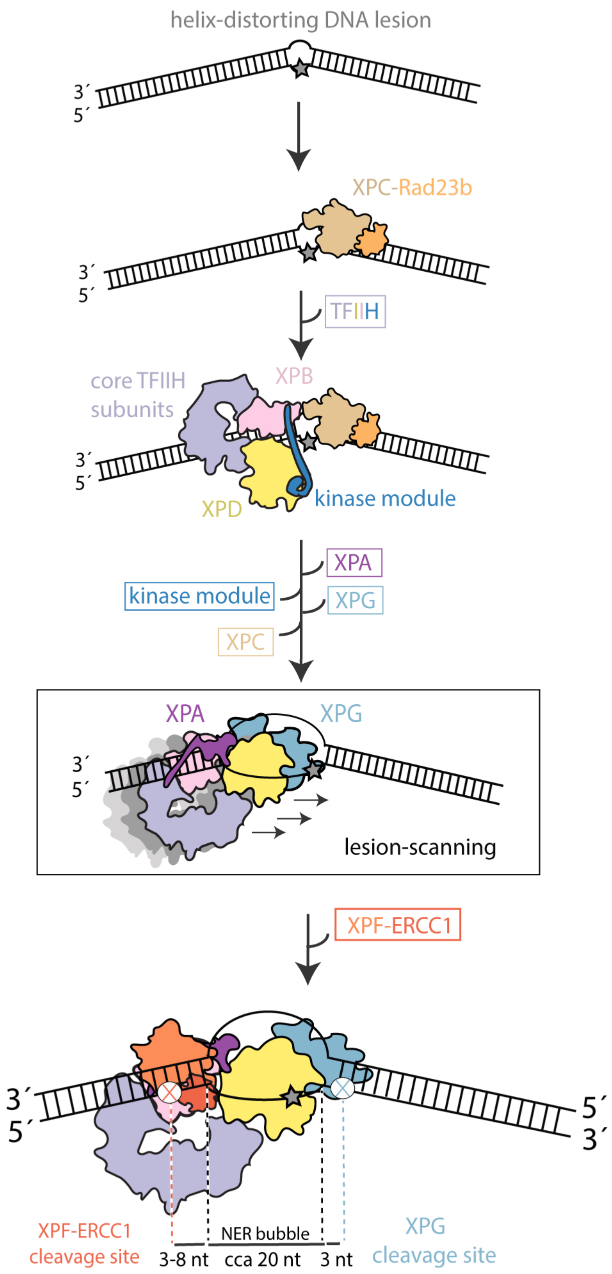

Supplementary Figure 8 | Schematic representation of the NER pathway highlighting new insights.

RPA was not included for clarity but it most likely engages the undamaged DNA strand of the repair bubble. Our structure suggests that XPA-XPD footprint on the DNA pre-defines the size of the excised DNA fragment during NER. See main text for more details.

Supplementary Table 1 | DNA sequences used in this study

Name	Sequence
H1	/56-FAM/TTCACCAGTGAGACGGGCAACAGC
H2	AAATCGCGCGTCTAGACTCA GCTCTGGCTGTTGCCCGTCTCACTGGTGAA/3BHQ_1/
H3	CGACAACGGGCAGAGTGACCACTT/36-FAM/
H4	/5IABkFQ/AAGTGGTCACTCTGCCCGTTGTCGGTCTCGACTCAGATCTGCGCGCTAAA
T1	GTCTTCTTTTAAACACTATCTTCCTGCTCATTTCTTTCTTCTTTCTTTTCTT
T2	/5IABkFQ/AAGAAAAGAAAGAAGAAAGAAATGAGCAGGAAGATAGTGTTTAAAAGAAGAC
T3	/56-FAM/TTCTTTTCTTTCTTCTTTCTTT

All synthetic DNA oligonucleotides were purchased from Integrated DNA Technologies.

Supplementary Table 2 | Cryo-EM data collection, refinement and validation statistics

	core TFIIH-XPA-DNA structure (EMD 4970) (PDB 6RO4)
**Data collection and processing**	
Magnification	130 000
Voltage (kV)	300
Electron exposure (e–/Å^2)	41
Defocus range (μm)	0.6-4.3
Pixel size (Å)	1.05
Symmetry imposed	C1
Initial particle images (no.)	1354997
Final particle images (no.)	227776
Map resolution (Å)<br>FSC threshold	3.5 (compsite map), 3.6 (globally refined map)
Map resolution range (Å)	3.1-7.7
**Refinement**	
Initial model used (PDB code)	Ab-initio in CryoSPARC
Model resolution (Å)<br>FSC threshold	3.4<br>0.5
Map sharpening *B* factor (Å^2)	Denoised map with flattened amplitude spectrum
Model composition<br>Non-hydrogen atoms<br>Protein residues<br>Ligands	 19824<br>2349<br>SF4, 6xZn
B factors (Å^2)<br>Protein<br>Ligand	 78.28<br>118.19
R.m.s. deviations<br>Bond lengths (Å)<br>Bond angles (°)	 0.006<br>0.948
Validation<br>MolProbity score<br>Clashscore<br>Poor rotamers (%)	 1.89<br>7.41<br>0.43
Ramachandran plot<br>Favored (%)<br>Allowed (%)<br>Disallowed (%)	 92.03<br>7.75<br>0.22

Supplementary Note 1 | DNA-bound TFIIH core at 3.6Å reveals structural etiology of Xeroderma pigmentosum (XP) and Trichothiodystrophy (TTD)

Mutations in the XPD helicase predominantly cause two human diseases with markedly different symptoms: Xeroderma pigmentosum (XP) and Trichothiodystrophy (TTD)[11,8], and in rare cases XP is additionally coupled with the Cockayne syndrome (CS)[12]. XP patients are extremely sensitive to UV-irradiation and readily develop skin cancer, while TTD patients suffer from mental and growth retardation, accelerated aging and decreased fertility[8]. Human XP and TTD mutations were mostly interpreted based on the comparison with the archaeal XPD homologues and modeled DNA trajectory through the helicase[13-15]. Since we solved the core TFIIH structure at higher resolution compared to previous models[4,16] and XPD helicase in our structure is bound to DNA (Fig. 4), we can precisely map human XP and TTD disease mutations and provide the structural basis for disease development.

Interestingly, the XP mutations[6-8] cluster around DNA in the ATPase lobe 2 of XPD (Supplementary Fig. 6a). Several of the affected residues (R683, R511 and S541) directly interact with the DNA backbone (Supplementary Fig. 6b). It is worth noting that mutations in R683, which binds the third to last nucleotide exiting XPD (Supplementary Fig. 6b), account for 64% of all XP mutations in XPD and for 55% of XP mutations in TFIIH overall[6]. An additional two mutations, D681N and Y542C, are found directly adjacent to DNA contacting residues: D681 forms a salt bridge with R683 and Y542 is located next to S541. Y542 stabilizes the loop containing S541 by interacting with V599, which is not possible when the bulky Y542 residue is replaced with a smaller cysteine (Supplementary Fig. 6b). Overall, the direct disruption of XPD-DNA contacts by these mutations, as seen in our structure, would impair the XPD helicase activity and NER, as also shown in vitro for S541R and R683 mutations[6]. Interestingly, another residue mutated in XP, R601, is located next to DNA, but the side chain of the residue points away from the DNA backbone (Supplementary Fig. 6b). However, R601 is placed next to the helicase motif Va and the long side chain of R601 forms a salt bridge with D655 and a cationic pair with H659, both of which are located in a helix carrying the helicase motif VI. Thus, substitution of R601 with a leucine or a much bulkier tryptophan in XP would disrupt these interactions and misalign helicase motifs, leading to XPD helicase impairment[6]. G602 is a direct neighbor of R601 and the substitution to a less flexible aspartate found in XP patients would probably have a similar disruptive effect as the R601 mutation (Supplementary Fig. 6b). In addition to disrupting the structural role of R601, G602D mutation also introduces a negative charge into the XPD DNA-binding groove, which may be particularly disruptive for XPD structure and function, and could explain why this mutation also leads to severe CS symptoms. Overall, most of the XP mutations in XPD directly target protein-DNA interactions which has a negative impact on XPD helicase activity and XPD function in NER, thus resulting in XP clinical features.

In contrast to XP, all but one TTD mutations[8] map to regions of our structure further away from the DNA trajectory (Supplementary Fig. 6a). Most of the mutations are located at the XPD-p44 interface. This interface is the main attachment point of XPD to the rest of the TFIIH core[17,18], but it is surprisingly small and could be particularly vulnerable to destabilizing mutations[6]. Interestingly, a single XPD helix (residues 711-725) is affected by four different TTD mutations (G713R, R722W, A725P and R487G) (Supplementary Fig. 6c). This helix is an important contact point between XPD and p44

because R722 and K718 in the helix form salt bridges with D75 and D73 located in a p44 loop (residues 68-76). The R722W mutation directly disrupts one of the two salt bridges. The G713R mutation likely destabilizes the helix because G713 is located in a hydrophobic pocket where a long arginine side chain cannot be accommodated nor neutralized. The R487G mutation can also be explained based on the structure. Even though R487 is distant from the helix in the primary structure, it engages in cation–π interaction with F728 located two residues away from the helix (Supplementary Fig. 6c). This R487-F728 interaction might keep the helix stacked against XPD and disruption of this interaction would likely displace the helix and weaken the XPD-p44 interface. An A725P substitution might have a similar disruptive effect on the helix because prolines have poor helix-forming propensities, and this substitution would also affect the neighboring R487-F728 interaction (Supplementary Fig. 6c). Two other destabilizing alanine to proline mutations (R592P and A594P) are located at the XPD-p44 interface (Supplementary Fig. 6a). Since XPD plays an important structural role during transcription initiation[16], a disruption of the XPD-p44 interface would cause a transcription defect and manifest in more severe clinical features as observed for TTD. Disruption of this interface would also have a negative effect on XPD function in NER because p44 stimulates XPD helicase activity[19], which would explain the NER defect in photosensitive forms of TTD[20].

The R658H and R658C TTD mutations do not map to the XPD-p44 interface but to the site between the ATPase lobes of XPD (Supplementary Fig. 6e). R658 participates in the extensive interaction network of charged residues between the lobes, which includes salt bridges with D240 located in the opposite lobe and D655 located in the same lobe. Thus, a mutation in R658 might destabilize the structure of XPD by disrupting contacts between the ATPase lobes, which may lead to transcription defects and TTD. R112H is the only TTD mutation in the vicinity of DNA. TFIIH carrying the R112H mutation is helicase dead and inactive in dual incision assay[6,21], and patient-derived cell lines are extremely UV-sensitive. However, the same mutation causes a mild defect in basal transcription in vitro and mild TTD symptoms in patients[6,21]. The helicase impairment of this XPD mutant is explained by our structure which shows that R112 directly binds the DNA phosphate backbone (Supplementary Fig. 6d). In addition, R112 physically couples DNA and the FeS cluster by interacting with the Fe-ligand C134 and the DNA backbone, which might be important for lesion recognition function of XPD[22]. Since R112 is located in front of the FeS cluster, the long side chain of the residue may shield the cluster from the reactive species in the solvent, as suggested before[23]. The R112 residue also interacts with F193 and Y192 and contributes to the structural stability of the FeS domain. It was indeed shown in yeast that this mutation results in the loss of the iron-sulfur cluster[24], which may destabilize the entire protein[14]. Structural destabilization of XPD would explain the effect of this mutation on transcription initiation and the manifestation of TTD clinical features. XPD and TFIIH destabilization by above mentioned mutations may also affect other TFIIH functions, such as gene transactivation[25], which contributes to the variety of symptoms of TTD patients.

An interesting case of a patient with a hybrid XP/TTD phenotype was reported relatively recently[9]. Aside from typical TTD clinical features such as brittle hair, the patient also developed skin cancer which is specific to XP. The sequence analysis of the XPD gene revealed 2 different mutations: A725P and Y18H (Supplementary Fig. 6a). We

already described how A725P mutation leads to destabilization of the XPD-p44 interface and a TTD phenotype. However, the side chain of the Y18 residue is located ~ 4Å away from the Q-motif/Q21 (Supplementary Figs. 4c,6a) which is predicted to bind ATP[3]. Y18H mutation might thus affect ATP hydrolysis and XPD helicase activity, which could explain the XP phenotype.

In summary, we mapped most of the XP and TTD mutations onto our XPD-DNA structure and we could visualize molecular interactions surrounding the affected residues. Thus, we provide a structural basis for how XP and TTD mutations impair XPD structure and function, and TFIIH integrity, thus leading to human disease development.

Supplementary References

1 Titov, D. V. *et al.* XPB, a subunit of TFIIH, is a target of the natural product triptolide. *Nature Chemical Biology* **7**, 182, (2011).
2 Buchan, D. W. A., Minneci, F., Nugent, T. C. O., Bryson, K. & Jones, D. T. Scalable web services for the PSIPRED Protein Analysis Workbench. *Nucleic acids research* **41**, W349-W357, (2013).
3 Fairman-Williams, M. E., Guenther, U.-P. & Jankowsky, E. SF1 and SF2 helicases: family matters. *Current opinion in structural biology* **20**, 313-324, (2010).
4 Greber, B. J. *et al.* The cryo-electron microscopy structure of human transcription factor IIH. *Nature* **549**, 414-417, (2017).
5 Coin, F. *et al.* Nucleotide Excision Repair Driven by the Dissociation of CAK from TFIIH. *Molecular cell* **31**, 9-20, (2008).
6 Dubaele, S. *et al.* Basal transcription defect discriminates between xeroderma pigmentosum and trichothiodystrophy in XPD patients. *Molecular cell* **11**, 1635-1646, (2003).
7 Cleaver, J. E., Thompson, L. H., Richardson, A. S. & States, J. C. A summary of mutations in the UV-sensitive disorders: xeroderma pigmentosum, Cockayne syndrome, and trichothiodystrophy. *Human mutation* **14**, 9-22, (1999).
8 Lehmann, A. R. The xeroderma pigmentosum group D (XPD) gene: one gene, two functions, three diseases. *Genes & development* **15**, 15-23, (2001).
9 Kralund, H. H. *et al.* Xeroderma Pigmentosum-Trichothiodystrophy overlap patient with novel XPD/ERCC2 mutation. *Rare diseases (Austin, Tex.)* **1**, e24932-e24932, (2013).
10 Kosinski, J. *et al.* Xlink Analyzer: software for analysis and visualization of cross-linking data in the context of three-dimensional structures. *Journal of structural biology* **189**, 177-183, (2015).
11 DiGiovanna, J. J. & Kraemer, K. H. Shining a light on xeroderma pigmentosum. *The Journal of investigative dermatology* **132**, 785-796, (2012).
12 Lehmann, A. R. XPD structure reveals its secrets. *DNA Repair (Amst)* **7**, 1912-1915, (2008).
13 Liu, H. *et al.* Structure of the DNA repair helicase XPD. *Cell* **133**, 801-812, (2008).
14 Fan, L. *et al.* XPD helicase structures and activities: insights into the cancer and aging phenotypes from XPD mutations. *Cell* **133**, 789-800, (2008).
15 Kuper, J., Wolski, S. C., Michels, G. & Kisker, C. Functional and structural studies of the nucleotide excision repair helicase XPD suggest a polarity for DNA translocation. *The EMBO journal* **31**, 494-502, (2012).
16 Schilbach, S. *et al.* Structures of transcription pre-initiation complex with TFIIH and Mediator. *Nature* **551**, 204, (2017).
17 Coin, F. *et al.* Mutations in the XPD helicase gene result in XP and TTD phenotypes, preventing interaction between XPD and the p44 subunit of TFIIH. *Nature genetics* **20**, 184-188, (1998).

18 Seroz, T., Perez, C., Bergmann, E., Bradsher, J. & Egly, J. M. p44/SSL1, the regulatory subunit of the XPD/RAD3 helicase, plays a crucial role in the transcriptional activity of TFIIH. *The Journal of biological chemistry* **275**, 33260-33266, (2000).
19 Coin, F., Oksenych, V. & Egly, J. M. Distinct roles for the XPB/p52 and XPD/p44 subcomplexes of TFIIH in damaged DNA opening during nucleotide excision repair. *Molecular cell* **26**, 245-256, (2007).
20 Hashimoto, S. & Egly, J. M. Trichothiodystrophy view from the molecular basis of DNA repair/transcription factor TFIIH. *Human molecular genetics* **18**, R224-230, (2009).
21 Botta, E. *et al.* Analysis of Mutations in the XPD Gene in Italian Patients with Trichothiodystrophy: Site of Mutation Correlates with Repair Deficiency, but Gene Dosage Appears to Determine Clinical Severity. *The American Journal of Human Genetics* **63**, 1036-1048, (1998).
22 Sontz, P. A., Mui, T. P., Fuss, J. O., Tainer, J. A. & Barton, J. K. DNA charge transport as a first step in coordinating the detection of lesions by repair proteins. *Proceedings of the National Academy of Sciences of the United States of America* **109**, 1856-1861, (2012).
23 Wolski, S. C. *et al.* Crystal structure of the FeS cluster-containing nucleotide excision repair helicase XPD. *PLoS biology* **6**, e149, (2008).
24 Rudolf, J., Makrantoni, V., Ingledew, W. J., Stark, M. J. R. & White, M. F. The DNA Repair Helicases XPD and FancJ Have Essential Iron-Sulfur Domains. *Molecular cell* **23**, 801-808, (2006).
25 Compe, E. *et al.* Neurological defects in trichothiodystrophy reveal a coactivator function of TFIIH. *Nature neuroscience* **10**, 1414-1422, (2007).
