# Supplementary material for: Structural basis of TFIIH activation for nucleotide excision repair
Source: Nat Commun. 2019 Jun 28;10:2885. doi: 10.1038/s41467-019-10745-5 (PMC6599211; doi:10.1038/s41467-019-10745-5)
Supplement: Supplementary file 2 — Description of Additional Supplementary Files [file 41467_2019_10745_MOESM2_ESM.pdf]

Description of Additional Supplementary Files

File Name: Supplementary Movie 1
Description: Structural changes in core TFIIH upon activation for DNA repair. The movie shows a morph between the core TFIIH conformations stabilized by the kinase module (Nogales lab) and by binding XPA and DNA (the conformations are described in more detail in the Supplementary Figure 5).

File Name: Supplementary Data 1
Description: Table of all BS3 crosslinks detected in core TFIIH-XPA-XPG-DNA. The file contains the table of all detected crosslinks and the original output of pLink 1.23 crosslinking spectra search.
