# Supplementary material for: Structural basis of TFIIH activation for nucleotide excision repair
Source: Nat Commun. 2019 Jun 28;10:2885. doi: 10.1038/s41467-019-10745-5 (PMC6599211; doi:10.1038/s41467-019-10745-5)
Supplement: Supplementary file 5 — Reporting Summary [file 41467_2019_10745_MOESM5_ESM.pdf]

nature research

Corresponding author(s): Patrick Cramer

Last updated by author(s): May 17, 2019

Reporting Summary

Nature Research wishes to improve the reproducibility of the work that we publish. This form provides structure for consistency and transparency in reporting. For further information on Nature Research policies, see Authors & Referees and the Editorial Policy Checklist.

Statistics

For all statistical analyses, confirm that the following items are present in the figure legend, table legend, main text, or Methods section.

n/a	Confirmed	
☐	☒	The exact sample size (*n*) for each experimental group/condition, given as a discrete number and unit of measurement
☐	☒	A statement on whether measurements were taken from distinct samples or whether the same sample was measured repeatedly
☒	☐	The statistical test(s) used AND whether they are one- or two-sided<br>*Only common tests should be described solely by name; describe more complex techniques in the Methods section.*
☒	☐	A description of all covariates tested
☒	☐	A description of any assumptions or corrections, such as tests of normality and adjustment for multiple comparisons
☐	☒	A full description of the statistical parameters including central tendency (e.g. means) or other basic estimates (e.g. regression coefficient) AND variation (e.g. standard deviation) or associated estimates of uncertainty (e.g. confidence intervals)
☒	☐	For null hypothesis testing, the test statistic (e.g. *F*, *t*, *r*) with confidence intervals, effect sizes, degrees of freedom and *P* value noted<br>*Give P values as exact values whenever suitable.*
☒	☐	For Bayesian analysis, information on the choice of priors and Markov chain Monte Carlo settings
☒	☐	For hierarchical and complex designs, identification of the appropriate level for tests and full reporting of outcomes
☒	☐	Estimates of effect sizes (e.g. Cohen's *d*, Pearson's *r*), indicating how they were calculated

Our web collection on statistics for biologists contains articles on many of the points above.

Software and code

Policy information about availability of computer code

Data collection	FEI EPU; ProteomeDiscoverer 2.1.0.81 (Thermo Scientific); pLink (v. 1.23)
Data analysis	Warp 1.0.6; RELION 3.0; CryoSPARC; COOT v. 0.8.9.1; PHENIX v. 1.14; Pymol v. 2.2.3; Chimera v. 1.12 ; GraphPad Prism version 7, XiNet, XlinkAnalyzer v. 1.1; Molprobity plugin (Phenix), PSIPRED; SWISS-Model (online server); MODELLER plugin in Chimera

For manuscripts utilizing custom algorithms or software that are central to the research but not yet described in published literature, software must be made available to editors/reviewers. We strongly encourage code deposition in a community repository (e.g. GitHub). See the Nature Research guidelines for submitting code & software for further information.

Data

Policy information about availability of data

All manuscripts must include a data availability statement. This statement should provide the following information, where applicable:

- Accession codes, unique identifiers, or web links for publicly available datasets
- A list of figures that have associated raw data
- A description of any restrictions on data availability

The electron density reconstruction and the final core TFIIH-XPA-DNA model were deposited with the Electron Microscopy Data Base (EMD-4970) and with the Protein Data Bank (PDB 6RO4). The crosslinking mass-spectrometry data have been deposited to the ProteomeXchange Consortium via the PRIDE partner repository with the dataset identifier PXD013947. Source data is relevant for Fig. 1a, Fig. 1b, Fig. 4d, Fig. 4e, Supplementary Fig. 1a, Supplementary Fig. 1c, Supplementary Fig. 1d, Supplementary Fig. 1f and Supplementary Fig. 2a.

Field-specific reporting

Please select the one below that is the best fit for your research. If you are not sure, read the appropriate sections before making your selection.

☒ Life sciences ☐ Behavioural & social sciences ☐ Ecological, evolutionary & environmental sciences

For a reference copy of the document with all sections, see nature.com/documents/nr-reporting-summary-flat.pdf

Life sciences study design

All studies must disclose on these points even when the disclosure is negative.

Sample size	No sample-size calculation was performed. All biological experiments were replicated two or more times.
Data exclusions	No data were excluded from the analysis.
Replication	All attempts at replication were successful.
Randomization	Samples were not allocated to groups.
Blinding	Investigators were not blinded as this is not applicable for methods used.

Reporting for specific materials, systems and methods

We require information from authors about some types of materials, experimental systems and methods used in many studies. Here, indicate whether each material, system or method listed is relevant to your study. If you are not sure if a list item applies to your research, read the appropriate section before selecting a response.

Materials & experimental systems

n/a	Involved in the study	
☐	☒	Antibodies
☐	☒	Eukaryotic cell lines
☒	☐	Palaeontology
☒	☐	Animals and other organisms
☒	☐	Human research participants
☒	☐	Clinical data

Methods

n/a	Involved in the study	
☒	☐	ChIP-seq
☒	☐	Flow cytometry
☒	☐	MRI-based neuroimaging

Antibodies

Antibodies used	RPB1 CTD anti phos ser5 3E8
Validation	The antibody was validated in the following study: Chapman et al., Science 2007

Eukaryotic cell lines

Policy information about cell lines

Cell line source(s)	Hi5 cells: Expression Systems, Tni Insect Cells in ESF921 media, Item 94-002F<br>Sf9 cells: ThermoFisher, Catalogue Number 12659017, Sf9 cells in Sf-900TM III SFM<br>Sf21 cells: Expression Systems, Sf21 insect cells in ESF921 medium, Item 94-003F
Authentication	Provided by commercial supplier (ThermoFisher and Expression Systems)
Mycoplasma contamination	Mycoplasma test was not required for used cell lines.
Commonly misidentified lines<br>(See ICLAC register)	No commonly misidentified cell lines were used.
